# Pathways to Aromatics in the Catalytic Pyrolysis of a Polyvinylchloride Model Compound Revealed by Operando Photoelectron Photoion Coincidence Spectroscopy

**DOI:** 10.1002/cssc.202500516

**Published:** 2025-05-26

**Authors:** Jovanni Cabana, Zihao Zhang, Zeyou Pan, Pawan Kumar, Xiangkun Wu, Andras Bodi, Gustavo A. Garcia, Yang Shen, Xintong Xiao, Hao Ma, Chen Huang, Chengyuan Liu, Long Zhao, Yang Pan, Zhongyue Zhou, Jeroen A. van Bokhoven, Patrick Hemberger

**Affiliations:** ^1^ Paul Scherrer Institute 5232 Villigen Switzerland; ^2^ Department of Chemistry and Applied Biosciences Institute for Chemical and Bioengineering ETH Zurich 8093 Zurich Switzerland; ^3^ Center for Renewable Carbon, School of Natural Resources University of Tennessee Knoxville TN 37996 USA; ^4^ Environment Research Institute Shandong University Qingdao 266237 China; ^5^ Synchrotron SOLEIL L’Orme des Merisiers St. Aubin,BP 48 91192 Gif‐sur‐Yvette France; ^6^ School of Mechanical Engineering Shanghai Jiao Tong University Shanghai 200240 China; ^7^ National Synchrotron Radiation Laboratory University of Science and Technology of China Hefei Anhui 230029 P. R. China; ^8^ School of Nuclear Science and Technology University of Science and Technology of China Hefei Anhui 230027 P. R. China

**Keywords:** aromatics formation, intermediates, operando, PEPICO, plastic pyrolysis

## Abstract

Dechlorination channels and pathways to olefins and aromatics in the catalytic pyrolysis of the polyvinylchloride (PVC) model compound 1,3‐dichlorobutane are revealed using operando photoelectron photoion coincidence (PEPICO) spectroscopy. Experimental and computational results agree that the primary pathway involves double dehydrochlorination producing 1,3‐butadiene and HCl. Minor radical channels are evidenced by the detection of chloromethyl, methyl, and propargyl radicals in thermal decomposition, while chlorine radicals are absent. HZSM‐5 zeolites lower the reaction temperature and facilitate 1,3‐butadiene association reactions producing C_5_–C_12_ olefins. Further reaction steps, detected experimentally and in part isomer‐selectively, mimic previously postulated cross‐linking pathways to aromatics in PVC catalytic pyrolysis. This study identifies C—C coupling as well as Diels–Alder dimerization of butadiene to yield polymethylated cyclopentadienes. These are central precursors to aromatics, for example, benzene, toluene, and xylenes (BTX). Ring expansion and contraction as well as transmethylation reactions are found to be dominant routes to aromatic products. The mechanisms during thermocatalytic conversion of PVC are applicable to other plastics and resemble the chemistry upon methanol‐ and methylchloride‐to‐hydrocarbon and aromatics conversion, which will inspire new strategies to enhance selectivity towards aromatics and mitigate coke formation.

## Graphical abstract

How are aromatics formed during catalytic PVC pyrolysis? By utilizing photoelectron photoion coincidence spectroscopy, the cross‐linking reaction mechanism is unveiled to proceed via butadiene dimerization and trimerization, yielding 5‐ or 6‐membered ring species, which are the precursors for benzene, toluene, and xylenes. These findings may open up new avenues to optimize plastic recycling or methanol‐to‐hydrocarbon processes.

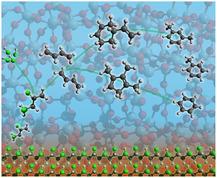

## 1 Introduction

**Scheme 1:**
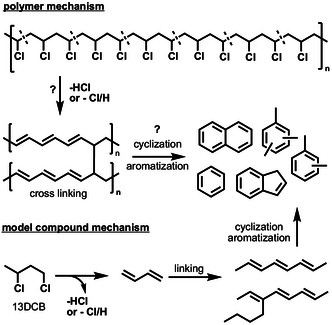
PVC catalytic pyrolysis mechanism proposal and model reaction (top). The dashed lines show the 13DCB moiety within PVC. Chemistry of the 13DCB model compound (bottom).

Polyvinylchloride (PVC) is, after polypropylene and polyethylene, the third most produced plastic in the world at more than 50 Mt y^−1^. Currently, only 10% of the globally produced plastics are recycled. As plastics demand is predicted to increase to 884 Mt y^−1^ in 2050, a circular economy requires that plastics recycling is addressed.^[^ ^1^ ^]^ PVC incineration releases toxic compounds, such as polychlorinated dioxins and furans; therefore, PVC waste is preferentially disposed of in landfills. This is far from sustainable and poses significant risks to human health and ecosystems, including the release of microplastics.^[^ ^2^ ^]^ While pyrolysis can degrade polymers to monomers, yielding fine chemicals, fuels, and aromatics, it is currently underutilized due to its complexity.^[^ ^3^ ^]^ Pyrolysis processes are usually optimized in a cook‐and‐look approach, where process parameters are varied until the optimum conversion and selectivity are achieved. However, understanding the reaction mechanism allows for targeted process optimization and may reveal shared reaction domains with other, better‐understood (catalytic) processes. Varied mechanisms have been proposed for PVC pyrolysis.^[^ ^4^, ^5^ ^]^ These follow three consecutive processes: 1) conversion of PVC into intermediates and HCl, 2) decomposition of intermediates into polyene chain and volatile gases, and 3) aromatics and chars formation.^[^ ^6^ ^]^ The first distinct stage involves dehydrochlorination of PVC between 250 and 320 °C^[^ ^7^ ^]^ with a 65% weight loss to yield polyenes. These polyene species are the precursors of the reforming process and step‐wise pyrolysis setups have been developed to avoid chlorinated products. Dehydrochlorination is usually performed at lower temperatures before the actual pyrolysis takes place.^[^ ^8^ ^]^ The second stage converts the dehydrochlorinated products (polyenes) to aromatics, alkenes, or waxes utilizing a catalyst to increase selectivities and reduce reaction temperatures. Conversion, product ratios (liquid versus gas) and selectivities depend on various parameters such as catalyst, plastic sample, temperature, and pyrolysis method.^[^ ^9^, ^10^, ^11^ ^]^ However, with the available experimental data and kinetic models, two fundamental questions remain unanswered as shown in **Scheme** **1**: 1) How is chlorine released? 2) By what mechanism are aromatics formed? Despite facile PVC dehydrochlorination to form HCl and an olefinic group, chlorobenzene and benzyl chlorides were also detected using FT‐IR spectroscopy in PVC conversion, indicating that chlorine may survive in aromatics^[^ ^12^, ^13^ ^]^ that are more difficult to dechlorinate.^[^ ^14^ ^]^ Chlorine radical loss reactions have been discussed, too, although atomic chlorine has not been observed.^[^ ^15^ ^]^

The production of benzenes, naphthalenes, or indenes is generally assumed to proceed either by cyclization and aromatization (Scheme 1) via cross‐linking or by Diels–Alder reactions of smaller dienes.^[^ ^13^, ^15^, ^16^, ^17^, ^18^ ^]^ In the absence of experimentally observed reactive intermediates and in the face of the overwhelming chemical complexity of primary PVC pyrolysis in the top‐down approach, an experimentally supported mechanism to aromatics remains elusive.^[^ ^12^, ^15^, ^17^ ^]^

In this study, we follow a bottom‐up approach to understand the PVC pyrolysis mechanism by investigating the (catalytic) pyrolysis of 1,3‐dichlorobutane (13DCB), which is the smallest model compound mimicking the structure of PVC. Furthermore, dehydrochlorination is usually anticipated to yield polyenes, which are subject to aromatics formation (Scheme 1, bottom). We achieve molecular‐level mechanistic understanding using photoelectron photoion coincidence (PEPICO) spectroscopy at in situ and operando conditions. PEPICO combines mass spectrometry and photoelectron spectroscopy correlating electrons and ions to isomer‐selectively detect reactive intermediates including radicals.^[^ ^19^, ^20^, ^21^, ^22^ ^]^ It is multiplexed, as numerous species can be detected at the same time, while also universal, since ionization is not limited by spectroscopic selection rules (see Supporting Information). PEPICO probes the gas phase above the catalyst surface and can thus distinguish surface‐confined from gas‐phase reactions,^[^ ^23^, ^24^ ^]^ with a sensitivity as low as 1 ppm in favorable cases.^[^ ^25^, ^26^ ^]^ High count rates may limit the dynamic range of the experiment, while the absence of strong origin transitions may impede isomer‐specific identification.

Although only a C_4_ model system is utilized, a rich network of olefins and aromatics is observed, which matches with the work of Pan et al.^[^ ^13^ ^]^ Our study gives new insights into the cross‐linking pathways, driven by dienes, which are relevant beyond PVC pyrolysis (Scheme 1) mechanisms, as they are also responsible for benzene, toluene, and xylene (BTX) formation in the methanol‐to‐hydrocarbons (MTH) process.^[^ ^27^, ^28^ ^]^

## 2 Results and Discussion

To capture reactive intermediates, the pyrolysis and catalytic pyrolysis of 13DCB are studied at short residence times, low pressures, and varying temperatures. Radical detection in microreactors (see Supporting Information)^[^ ^29^, ^30^ ^]^ involves expanding the effluent gas into vacuum to form a molecular beam, which creates a snapshot of the chemistry due to a rapid transfer of the beam into the operando PEPICO spectrometer.

**Figure 1:**
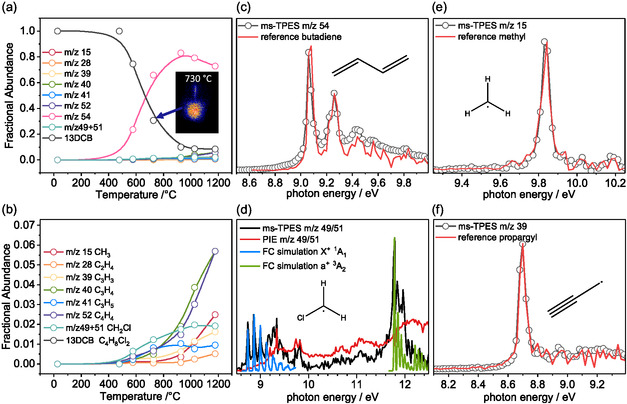
a) Fractional abundances of the 13DCB pyrolysis products obtained from mass spectra recorded at 10.5 eV photon energy (Figure S2, Supporting Information). Pyrolysis is initiated at 500 °C leading to 1,3‐butadiene (*m*/*z* 54). b) Zoom of low selectivity products. c–f) Photoion mass‐selected threshold photoelectron spectra of reaction products identified using reference spectra and Franck–Condon spectral modeling. Conditions: 20 sccm gas flow of 0.1% 13DCB in argon into the 1 mm iD Chen‐type microreactor (RT–1180 °C, a), *p* = 10–20 mbar at a residence time of 20–50 μs. Intermediate and product detection using the operando PEPICO setup at the Swiss Light Source.

13DCB (*m*/*z* 127/129) starts to decompose in the microreactor at around 530 °C (**Figure** **1**a,b), leading to the formation of *m*/*z* 54, which can be assigned as 1,3‐butadiene based on the agreement of the ms‐TPES (Figure 1c) with the reference spectrum in the PEPISCO database.^[^ ^20^ ^]^ The characteristic peaks at *m*/*z* 36/38 at 13.2 eV photon energy (Figure S2, Supporting Information) evidence HCl formation already below 530 °C. Further pyrolysis intermediates and products are observed and assigned as methyl (*m*/*z* 15, Figure 1e), propargyl (*m*/*z* 39, Figure 1f), and chloromethyl radicals (*m*/*z* 49/51, Figure 1d). Franck–Condon spectral modeling of the vibrational fingerprint of the singlet (${\tilde{X}}^{+}$ ^1^A_1_, blue line, Figure 1d) and triplet (${\tilde{a}}^{+}$ ^3^A_2_, green line, Figure 1d) cation states coupled with the G4 adiabatic ionization energy (AIE_G4_ = 8.74 eV) reproduces the fundamental transition observed at 8.74 eV and the spectrum of chloromethyl well. Chlorine radicals (AIE_exp_ = 12.96764 eV)^[^ ^31^ ^]^ and molecular chlorine (AIE_exp_ = 11.481 eV)^[^ ^32^ ^]^ were not detected (Figure S2, Supporting Information). Thus, HCl and CH_2_Cl formation represents the major unimolecular thermal dechlorination channels of 13DCB.

**Scheme 2:**
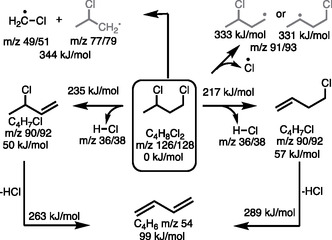
Reaction mechanism of the unimolecular decomposition of 1,3‐dichlorobutane (13DCB) calculated at G4 level of theory. Barrier heights are given on top of the reaction arrow, while 0 K reaction energies are shown with the products. The products are at the highest energy in the two radical channels (top). Species in gray were not directly observed.

Ethylene (*m*/*z* 28), allyl (*m*/*z* 41), vinylacetylene (*m*/*z* 52), and chlorobutenes (*m*/*z* 90/92) are also assigned by their ms‐TPES (Figure S3, Supporting Information). Although chlorobutenes are trace intermediates due to further rapid dehydrochlorination to butadiene, and their signal is perturbed by chlorobutene ions as dissociative ionization products of 13DCB, their presence could be unambiguously established thanks to ion velocity map imaging (VMI, inset Figure 1a, S4, Supporting Information). 1,3‐Butadiene (*m*/*z* 54) and chloromethyl (*m*/*z* 49/51) appear at the same temperature, while methyl (*m*/*z* 15) and propargyl (*m*/*z* 39) signals are only seen at higher ones, accompanied by decreasing 1,3‐butadiene intensity. This suggests that butadiene and chloromethyl are primary, while methyl and propargyl are secondary pyrolysis products (see Figure S5, Supporting Information).^[^ ^33^ ^]^ The potential energy surface for 13DCB decomposition at G4 level of theory (Figure S5, Supporting Information) shows two parallel HCl‐loss channels by removing the 1‐ or 3‐chlorine at similar barriers of 217 and 235 kJ mol^−1^ (**Scheme** **2**), respectively. 3‐ and 4‐Chloro‐1‐butene decompose further by a second dehydrochlorination to yield 1,3‐butadiene.

Chloromethyl and 2‐chloropropyl form in parallel at a 100 kJ mol^−1^ higher activation energy (Scheme 2, Figure S5, Supporting Information). The latter is subject to dehydrochlorination to allyl, in line with the simultaneous appearance of allyl radicals (Figure 1b). Although these channels are more endothermic, the reaction proceeds over a loose transition state, which makes them entropically favored at higher temperatures. Chlorine radical loss from 13DCB is similarly endothermic, but was not observed, likely because the related dehydrochlorination reaction is favored and HCl is released instead. Secondary products of 1,3‐butadiene are discussed in the supporting information (Figure S5, Supporting Information).

Thus, the unimolecular decomposition of 13DCB yields mostly 1,3‐butadiene and HCl, which compares well with the PVC pyrolysis experiments of Pan et al.^[^ ^13^ ^]^ Minor radical products such as chloromethyl, methyl, allyl, and propargyl are observed at higher temperatures. Methyl and propargyl are associated with transmethylation^[^ ^34^ ^]^ and coke formation channels,^[^ ^35^ ^]^ respectively, and accelerate catalyst deactivation. Atomic chlorine was not detected, and only aromatic precursors were observed due to the short residence time, limiting bimolecular chemistry.

**Figure 2:**
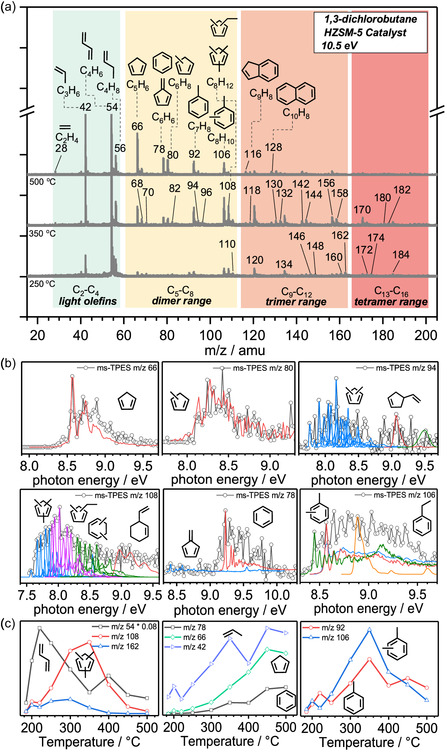
a) Catalytic pyrolysis mass spectra of 13DCB over HZSM‐5. In addition to 1,3‐butadiene, we found dimer‐, trimer‐, and tetramer‐range intermediates and products. b) Isomers are identified based on the ms‐TPES, Franck–Condon spectral modeling, and reference spectra. c) Peak intensities as function of temperature. Conditions: 30 sccm total flow rate, 0.3% 13DCB in argon, T = RT–500 °C, preactor inlet = 1.2–1.3 bar, 25 mg HZSM‐5. Operando PEPICO setup at DESIRS beamline at Soleil Synchrotron.

The reaction temperature to achieve full conversion to 1,3‐butadiene and HCl is reduced to 180 °C upon utilizing HZSM‐5 (Si/Al ratio 25, **Figure** **2**a), while enabling also diene C—C coupling (cross‐linking). Mass spectra (Figure 2a and S6,S7, Supporting Information) show the selectivity of the process and the intermediate and product abundances. Catalytic dehydrochlorination dominates, and radical formation channels are suppressed quantitatively.

The C_*x*_ species in the mass spectrum (Figure 2, S7, Supporting Information) exhibit peak progressions spaced by *m*/*z* 14 (CH_2_), indicating either stepwise methyl addition reactions^[^ ^27^, ^36^ ^]^ or butadiene oligomerization and cracking. However, closer inspection of the signal dependence on the temperature reveals that *m*/*z* 106–108 (dimers) and 156–162 (trimers) are observed with a higher abundance at 250 °C (Figure 2a,c, S8, Supporting Information), while butadiene is consumed and lighter olefins and aromatics between *m*/*z* 66 and 106 are formed in lower abundances (Figure 2a,c, S8‐11, Supporting Information). Consequently, butadiene oligomerizes to produce *m*/*z* 108 and 162 with maximum selectivity to these species at around 300 °C (Figure 2c, S8, Supporting Information). These channels model the cross‐linking pathways observed in PVC pyrolysis (Scheme 1), hypothesized to be responsible for aromatics formation.^[^ ^4^, ^15^, ^16^, ^18^, ^37^ ^]^ To assess whether surface‐bound chlorine or HCl influences the mechanism of butadiene generated by dehydrochlorination of 13DCB, we also investigated the catalytic conversion of 1,3‐butadiene with photoionization mass spectrometry and directly compared it to the mass spectra of 13DCB (Figure S12a,b, Supporting Information). We found that the light‐off temperatures are similar at around 200 °C, where dimers, trimers, and tetramers of 1,3‐butadiene are observed. In particular, the large abundance of *m*/*z* 108 in the intermediate temperature range and its decomposition at higher temperatures are independent of the reactant used. Minor differences persist in the formation of *m*/*z* 90/92, which are attributed to the presence of trace amounts of chlorobutenes. This confirms the dimer's and trimer's temperature dependence (Figure S12b, Supporting Information) and the spectator role of chlorine during the butadiene coupling reaction. However, Ibáñez et al.^[^ ^38^ ^]^ reported that HCl can play a role in the catalyst stability at temperatures exceeding 450 °C. In agreement with other groups,^[^ ^39^, ^40^, ^41^ ^]^ they identified dealumination of the catalyst due to AlCl_3_ formation, resulting in reduced catalyst activity after regeneration.

**Scheme 3:**
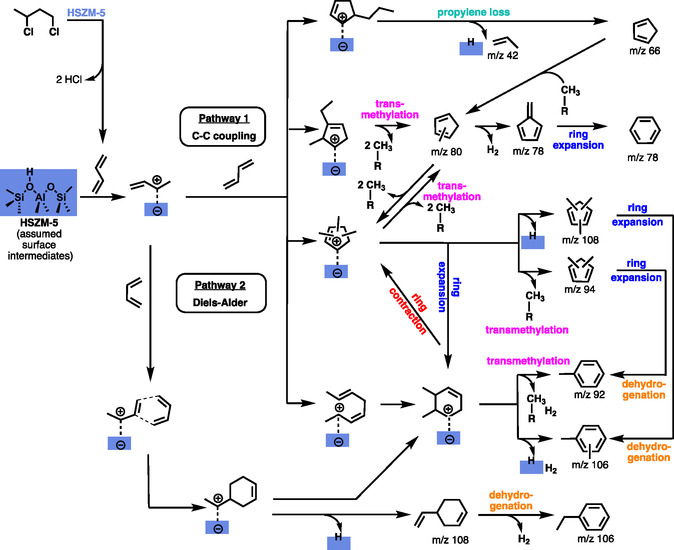
Dehydrochloriantion of 13DBC to 1,3‐butadiene and dimerization mechanisms over HZSM5. Assumed surface species are color coded in blue, while the black intermediate and products are observed after desorption from the catalyst.

Above 300 °C, the selectivities of lighter species, for example, *m*/*z* 106 by hydrogen transfer or *m*/*z* 156–160, increase, while the dimer and trimer signals decline in both 13DCB (Figure 2c, S8, Supporting Information) and 1,3‐butadiene conversion (Figure S12b, Supporting Information). In addition, larger olefins and aromatics, such as propylene (*m*/*z* 42), butylenes (*m*/*z* 56), cyclopentadiene (*m*/*z* 66), benzene (*m*/*z* 78), methylcyclopentadiene (*m*/*z* 80), toluene (*m*/*z* 92), dimethylbenzene (*m*/*z* 94), and xylenes/ethylbenzene (*m*/*z* 106), are also assigned based on their ms‐TPES and PI spectra (Figure 2b, S9‐11, Supporting Information). The C_3_–C_8_ species in our model system reflect the HZSM‐5 catalyzed pyrolysis of mixed plastics by Marino et al. and Pan et al.^[^ ^13^, ^18^ ^]^ These species are formed above 250 °C, when butadiene oligomers crack on the catalyst surface. This is illustrated by the increasing cyclopentadiene (*m*/*z* 66) and propylene (*m*/*z* 42) signals, while the butadiene dimer (*m*/*z* 108, Figure 2c, S8, Supporting Information) simultaneously decreases (**Scheme** **3**). This raises the question of the identity of the dimer species and the mechanism of olefins and aromatic formation. The *m*/*z* 80 and 94 (Figure 2b) peaks are assigned to methylated cyclopentadienes, which are also observed in the methylchloride/methanol‐to‐hydrocarbons reaction (MCTH/MTH)—and linked to aromatics formation.^[^ ^27^, ^36^ ^]^ In general, 5‐membered rings are more stable than their acyclic counterparts (Figure S13, Table S1, Supporting Information). Thus, it is reasonable to start fitting the *m*/*z* 108 ms‐TPES by trimethylcyclopentadiene isomers. This preliminary fit reproduced the low‐energy part of the TPES and could be improved by including FC simulations of ethylmethylcyclopentadienes and dimethylcyclohexadienes. Moreover, the FC simulation of the Diels–Alder adduct, 5‐vinylcyclohex‐1‐ene, shows a structured band located at 9 eV (AIE_G4_ = 8.99 eV), which overlaps with a low‐intensity band in the experimental spectrum (Figure 2b).^[^ ^42^ ^]^ The weak signal may be explained by fast isomerization to the more stable cyclic isomers (see below). Overall, numerous quasi‐isoenergetic methyl‐ and ethyl‐substituted five‐ and six‐membered ring species may contribute to the C_8_H_12_ isomers (*m*/*z* 108, Table S1, Supporting Information). The C_8_H_12_ signal declines above 350 °C (Figure S8, Supporting Information) and is virtually zero at 450 °C, while *m*/*z* 106 and 92 exhibit high abundances. This suggests that the dimer (*m*/*z* 108) is the precursor to xylenes and ethylbenzene (*m*/*z* 106) via hydrogen transfer reactions (Figure S13, Supporting Information).

Scheme 3 shows routes to five‐ and six‐membered ring species starting from butadiene, which forms a carbenium ion or an alkoxy species on HZSM‐5, in analogy to the results from the literature.^[^ ^43^, ^44^ ^]^ These adsorbed butadiene species couple with each other, modeling the cross‐linking mechanism during PVC pyrolysis. Our study detects only the desorbed species, and thus, the intermediates shown only represent plausible surface species (blue box) inferred from the observed intermediates and products. Surface‐catalyzed cyclization (Scheme 3, Pathway 1) and isomerization yields dimethyl‐substituted cyclohexenes as well as methyl‐, ethyl‐, and propyl‐substituted cyclopentene derivatives, which, after desorption, dominate the *m*/*z* 108 signal (Figure 2a). Hydrogen‐ or methyl‐transfer drive the chemistry of trimethyl‐, dimethyl‐, or methylcyclopentadiene (*m*/*z* 108, 94, and 80), respectively (Scheme 3). These methylated five‐membered ring species can expand and dehydrogenate to afford xylenes (*m*/*z* 106) and toluene (*m*/*z* 92, Figure S13, Supporting Information), making them viable rate‐limiting intermediates to stable aromatics.

Alternatively, terminal C—C addition intermediates can ring close to a C_3_‐substituted cyclopentane (Scheme 3 top trace) intermediate on the surface, which is subject to propylene and cyclopentadiene formation, as supported by the peaks observed at *m*/*z* 66 and 42 (Figure 2).

In addition, 6‐rings may also form via surface catalyzed [4 + 2] Diels–Alder cycloaddition (Scheme 3, Pathway 2), as indicated by the small band at 9 eV, which is assigned to vinylcyclohexene (Figure 2b *m*/*z* 108). Subsequent dehydrogenation (Scheme 3, bottom trace) should produce ethylbenzene, which is only observed in low quantities according to the ms‐TPES (Figure 2b, *m*/*z* 106). This may point to a dynamic equilibrium between six‐ and five‐membered ring species, which may be shifted to five‐membered species as seen by their high abundance in the ms‐TPES. This is further confirmed by observations from Chizallet et al. who investigated ring contraction and ring expansion reactions of ethylcyclohexane over zeolites.^[^ ^45^ ^]^

Only traces of ethylene are observed (Figure S7, Supporting Information), which means that C_2_ loss from the C_8_ dimer is a marginal process. As C_6_ methylcyclopentadienes are clearly detected, a stepwise demethylation (indicated as R‐CH_3_ in Scheme 3) is likely responsible for their formation instead of direct ethylene production. Hydrogen transfer affords fulvene, the precursor of benzene (Scheme 3). These methyl species from polymethylated cyclopentadienes or benzenes (R‐CH_3_ Scheme 3) are available for C—C coupling to yield xylenes from toluene and benzene at higher temperatures, which shows again the relation of the butadiene dimerization with the methylchloride and methanol to hydrocarbons chemistries. The butadiene dimer mechanism can, thus, lead to species with *m*/*z* > 108, such as *m*/*z* 120, following methylation. However, the disproportionately high abundance of trimethylbenzenes, polymethylated naphthalenes, and indenes calls for a further pathway, the trimer reaction route as discussed in the supporting information (Figure S14, Supporting Information). The products of this route are potent precursors to larger polycyclic aromatic hydrocarbons (PAHs) and coke, accelerating catalyst deactivation.

Chlorinated olefins and aromatics^[^ ^13^ ^]^ only play a minor role under our conditions, which can be rationalized by the rapid dehydrochlorination of 13DCB at low temperatures. The coupling of butadiene to yield olefins and aromatics is likely enabled over HZSM‐5 due to its high acidity (Figure S1, Supporting Information). These observations allowed us to reveal the first steps of the cross‐linking mechanism to produce BTX during PVC and other plastic pyrolysis processes, driven by dienes.^[^ ^18^ ^]^

## 3 Conclusion

In summary, we have unveiled dechlorination reactions and the mechanism of olefin and aromatics formation during (catalytic) pyrolysis of the PVC model compound 1,3‐dichlorobutane by detection of stable and reactive intermediates and computations. The initial reaction is a double dehydrochlorination yielding 1,3‐butadiene. While chlorine atoms could not be observed, chloromethyl, methyl, and propargyl radicals were detected and may participate in catalyst deactivation and transmethylation reactions at higher temperatures.

HZSM‐5 lowers the reaction temperature to afford mostly butadiene and HCl, while no radicals were desorbed from the surface. The reactivity of butadiene over zeolitic active sites enables C—C coupling, cracking, and transmethylation reactions to afford C_5_–C_12_ species to unveil the cross‐linking mechanism during catalytic PVC pyrolysis, ultimately leading to aromatics formation.^[^ ^13^, ^17^ ^]^ Experimental detection of butadiene dimers (C_8_) evidences acid‐catalyzed Diels–Alder and terminal C_4_ coupling (Pathway 1 and 2 in Scheme 3) reactions, giving rise to five‐ and six‐membered ring intermediates, which are the precursors of BTX. Species heavier than C_8_ are formed via methylation and mostly by butadiene trimerization to yield trimethylbenzenes, indenes, and naphthalenes. Detection of PAHs and their precursors, such as propargyl (C_3_H_3_), rationalize coke formation channels, deactivating the catalyst.

The results reveal rate‐limiting reaction intermediates and pathways to stable aromatics and PAHs during PVC catalytic pyrolysis, which are also applicable to PP and PE thermocatalytic conversion due to the high reactivity of diolefins.^[^ ^46^ ^]^ These mechanistic insights illustrate new opportunities to fine tune catalysts, reaction temperatures, and other process conditions to enhance product selectivity toward aromatics and catalyst stability during PVC reutilization. Suppression of trimerization intermediates may reduce coke precursor formation and extend the lifetimes of zeolite catalysts. Furthermore, our diene dimerization mechanism can also explain pathways to aromatics in MTH and MCTH reactions, justifying the universal applicability of our discovered chemistry for a targeted optimization of these processes.

## Conflict of Interest

The authors declare no conflict of interest.

## Supporting information

Supplementary Material

## Data Availability

The data that support the findings of this study are available in the supplementary material of this article. The derived data can be obtained from: https://doi.org/10.5281/zenodo.15340641.
