# Supplementary material for: Pathways to Aromatics in the Catalytic Pyrolysis of a Polyvinylchloride Model Compound Revealed by Operando Photoelectron Photoion Coincidence Spectroscopy
Source: ChemSusChem. 2025 May 26;18(14):e202500516. doi: 10.1002/cssc.202500516 (PMC12270361; doi:10.1002/cssc.202500516)
Supplement: Supplementary file 1 — Supplementary Material [file CSSC-18-e202500516-s001.pdf]

Pathways to Aromatics in the Catalytic Pyrolysis of a PVC Model Compound Revealed by Operando Photoelectron Photoion Coincidence Spectroscopy

Jovanni Cabana,[a,b] Zihao Zhang,[a,c] Zeyou Pan,[a,b] Pawan Kumar,[a] Xiangkun Wu,[a,d] Andras Bodi,[a] Gustavo A. Garcia,[e] Yang Shen,[f] Xintong Xiao,[f] Hao Ma,[g] Chen Huang,[g] Chengyuan Liu,[g] Long Zhao,[g,h] Yang Pan,[g] Zhongyue Zhou,[f] Jeroen A. van Bokhoven,[a,b] and Patrick Hemberger[a]*

[a] J. Cabana, Prof. Dr. Z. Zhang, Dr. Z. Pan, P. Kumar, Dr. A. Bodi, Dr. X. Wu, Prof. Dr. J. van Bokhoven and Dr. P. Hemberger, Paul Scherrer Institute, 5232 Villigen, Switzerland. E-mail: patrick.hemberger@psi.ch

[b] J. Cabana, Dr. Z. Pan, Prof. Dr. J. A. van Bokhoven, Institute for Chemical and Bioengineering, Department of Chemistry and Applied Biosciences, ETH Zurich, 8093 Zurich, Switzerland

[c] Prof. Dr. Z. Zhang Center for Renewable Carbon, School of Natural Resources, University of Tennessee, Knoxville, TN 37996, USA

[d] Dr. X. Wu, Environment Research Institute, Shandong University, Qingdao 266237, China

[e] Dr. G. A. Garcia, Synchrotron SOLEIL, L'Orme des Merisiers, St. Aubin, BP 48, 91192 Gif-sur-Yvette, France

[f] Dr. Y. Shen, X. Xiao, Prof. Dr. Z. Zhou, School of Mechanical Engineering, Shanghai Jiao Tong University, Shanghai 200240, China

[g] H. Ma, C. Huang, Prof. Dr. L. Zhao, Prof. Dr. Y. Pan, National Synchrotron Radiation Laboratory, University of Science and Technology of China, Hefei 230029, Anhui, P. R. China

[h] Prof. Dr. L. Zhao, School of Nuclear Science and Technology, University of Science and Technology of China, Hefei 230027, Anhui, P. R. China

1 Experimental and Computational Section

1.1 Samples

1,3-dichlorobutane (99%) was purchased from Sigma-Aldrich. The HZSM-5 (Si/Al=25) catalyst, was purchased from Zeolyst International. HZSM-5 was calcined in static air at 550 °C for 6 h using a heating rate of 2 °C min^{-1} before use.

1.2 Computational details

Gaussian 16 rev. A.03 and C.01[1] was utilized to perform quantum chemical calculations. Optimized geometries and vibrational frequencies were computed at the B3LYP/6-311++G(d,p) level and used in the Franck–Condon (FC) simulations. The obtained stick spectra are convolved with a Gaussian function with a full width at half maximum (fwhm) of 30–80 meV and fitted to the experimental ms-TPES for isomer-specific assignment. Adiabatic ionization energies for products and intermediates were evaluated using G4 or CBS-QB3 theory. These composite methods include a sequence of calculations at different levels of theory starting from geometry optimizations and vibrational analysis and include energy corrections and empirical parameters to achieve chemical accuracy of 1 kcal/mol.[2] Somers and Simmie [3] have found that G4 provides thermochemical parameters with better than chemical accuracy. To screen ionization energies, we used the computationally less expensive CBS-QB3 composite method. To fit the FC simulations of the m/z 108 isomers to the ms-TPES data we allowed a confidence

interval of ± 100 meV from the CBS-QB3 calculated adiabatic ionization energies, which has been found to be a valid approach in previous isomer screening tests.[4] Reaction coordinates and transition states were calculated via Berny search or using STQN methods and further refined using G4 or CBS-QB3.[5] Vibrational analysis was carried out to confirm transition states (i.e. reaction paths) or true minima on the potential energy surface.

1.3 Pyrolysis – photoelectron photoion coincidence (PEPICO) spectroscopy at Swiss Light Source

Pyrolysis experiments were carried out at VUV (x04db) beamline of the Swiss Light Source (SLS), located at Paul Scherrer Institute (PSI) in Villigen (Switzerland). Only a brief description is given here as most methodologies have already been discussed in the literature.[6] The 1,3-dichlorobutane (13DCB) sample was diluted using argon as a carrier gas to obtain ca. 0.1 % concentration in the gas phase. 20 sccm of this mixture was expanded though a 100–200 μm nozzle in a heated Chen-type reactor (40 mm length, 25 mm heated zone, 1 mm inner diameter), operated in the RT–1300 °C temperature range at an inlet pressure of 10–20 mbar, resulting in a residence time of ca. 20–50 μs. The effluent gas is rapidly expanded into a vacuum chamber (10^{-5} mbar) forming a molecular beam (MB, see **Scheme S1**). The MB is skimmed (2 mm Beam Dynamics skimmer) and travels towards the ionization volume of the CRF-PEPICO endstation.[6b] Tunable vacuum ultraviolet VUV synchrotron radiation was produced by a bending magnet and guided by a collimating mirror towards a monochromator, equipped with a 150 lines/mm grating. The dispersed light is focused onto the exit slit (200 μm) located in the gas filter, which is used to suppress the higher order radiation, leading to a resolving power of 1500. The VUV light is intersected with the skimmed molecular beam in the ionization volume to generate both a photoelectron and a photoion. In PEPICO, the charged particles are accelerated in opposite directions and detected in delayed coincidence to mass analyze the cations based on their time of flight. When charged particles are velocity map imaged (VMI) onto position sensitive detectors from Roentdek (DLD40), this also permits us to distinguish the molecular beam components from the room temperature background and to plot mass spectra and photoion mass-selected threshold photoelectron spectra (ms-TPES). The latter ones were obtained by subtracting the false coincidence and the hot electron backgrounds.[7]

1.4 Catalytic Pyrolysis – photoelectron photoion coincidence (PEPICO) spectroscopy at Synchrotron Soleil

The same sample delivering system, as described in the previous chapter, was applied to transport a mixture of 0.1–0.3 % 13DCB in argon at a flow rate of 30 sccm towards the catalytic reactor installed at the DESIRS beamline[8] at Synchrotron Soleil (**Scheme S1**). Only a brief description of the reactor[9] and beamline is given here. A quartz reactor was filled with ca. 25 mg catalyst, held between two quartz wool beds (4 mm diameter, 15 mm length). A tubular heater (*Heatwave Labs.*) surrounds the reactor and is controlled by a regulator (*Heatwave Labs.*) to reach isothermal conditions of the bed, as measured by a type K thermocouple. The catalysis source was mounted on the three-axis manipulator

of the SAPHIRS endstation located in the expansion chamber. After double skimming, a small portion of the MB reaches the center of the DELICOUS III[10] spectrometer, where it is intersected with VUV radiation as generated by the OPHELIE variable polarization undulator. Under the measured conditions a combined photon and electron energy resolution of 8 meV is achieved. Photoelectrons and ions are accelerated onto position sensitive detectors to obtain both mass spectra as well as photoion mass-selected threshold photoelectron spectra. The latter were corrected for false coincidences and hot electron contamination. In a typical test reaction, the catalyst was pretreated in argon for 30 minutes at 500 °C and thereafter cooled down to around 180 °C.

1.5 Catalytic Pyrolysis – photoionization mass spectrometry (PIMS) at Hefei Light Source

Photoionization mass spectrometry measurements of 1,3-butadiene over HZSM-5 were performed at the National Synchrotron Radiation Lab (NSRL) in Hefei (China) at beamline BL03U.[11] The mass spectrometer and beamline were described in detail in the literature.[12] A mixture of 1,3-butadiene in argon (0.1%) at a flow rate of 50 sccm was sent in a quartz tube reactor equipped with HZSM-5 held by a sintered quartz piece and wool.[13] The reaction was carried out at a pressure of 7 mbar in the RT–600 °C temperature range. The effluent gas forms a molecular beam through a 350 μm quartz nozzle, which was skimmed and fed into the gas inlet of the mass spectrometer. Photoionization mass spectra were taken at fixed photon energies and different temperatures.

1.6 Comparison of the used detection methods and reactors

a)

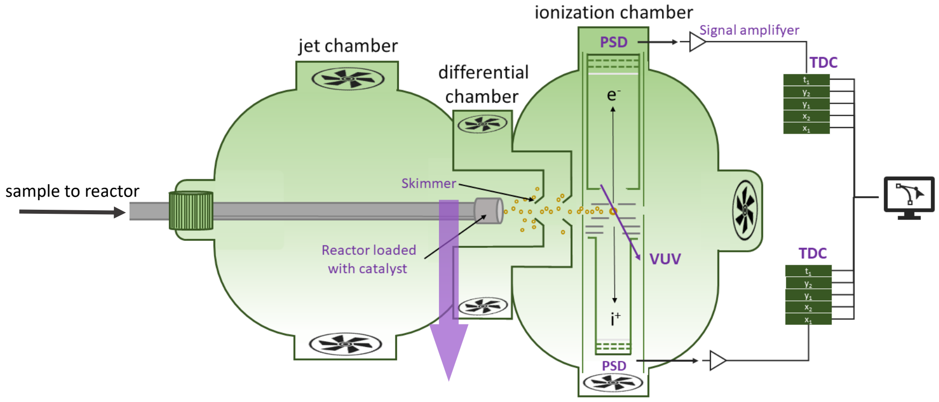

b)

	type	diameter	pressure	temperature	residence time
Photoelectron Photoion Coincidence Spectroscopy (PEPICO)	Chen-type pyrolysis reactor	1 mm	10-20 mbar	≤ 1300 °C	20-50 μs
Photoelectron Photoion Coincidence Spectroscopy (PEPICO)	cylindrical reactor	4 mm	0.3- 1.3 bar	≤ 600 °C	< 10 ms
Photo Ionization Mass Spectrometry (PIMS)	cylindrical reactor	6 mm	7 mbar	≤ 600 °C	< 10 ms

Scheme S1 a) Graphical representation of the operando PEPICO setup at SOLEIL, which is schematically similar with the one from SLS. **b)** compares the reactors used.

While both at SLS and Soleil Synchrotron operando PEPICO spectroscopy is utilized (**Scheme S1a**), the Hefei Setup is capable of measuring photoionization mass spectra (PIMS). The conceptual differences of these methods are summarized in the literature and are mainly based on detection of both electrons and ions or ions only.[14] A comparison of the different reactors used during the experiments is presented in **Scheme S1b**, which details the type, diameter, pressure and temperature range, as well as the residence times used during the reactions. The Chen-type reactor is designed for short residence times[15], with high radical detection capabilities and a small volume for the catalyst due to the 1 mm inner diameter. The other two cylindrical reactors can be equipped with more catalyst, and they have slight differences in their operation due to their respective pumping schemes. The reactor operated with the PEPICO system at Soleil Synchrotron measures the pressure at the entrance of the catalyst bed, which stagnates through the bed, while the reactor used for the PIMS measurements regulates the pressure in the catalysis chamber via a butterfly valve, leading to a uniform pressure profile. Despite the difference in the operation of the latter two reactors, the catalysts tests show similar selectivities as shown in **Figure S12**.

1.7 Catalyst Characterization

catalyst	HZSM-5
**Average poor diameter/ Å**	5.4–5.6
V_{micro} **(cm³/g)**	0.15
S_{BET} **(mmol/g)**	364
**BAS (mmol/g)**	0.33
**LAS (mmol/g)**	0.064

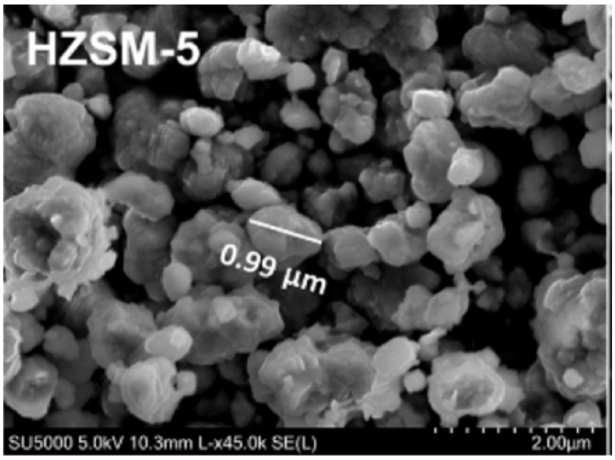

Figure S1 HZSM-5 characterization and TEM image. Data and figure were partially taken from ref. [16] under the CC-BY license.

2. Experimental Results

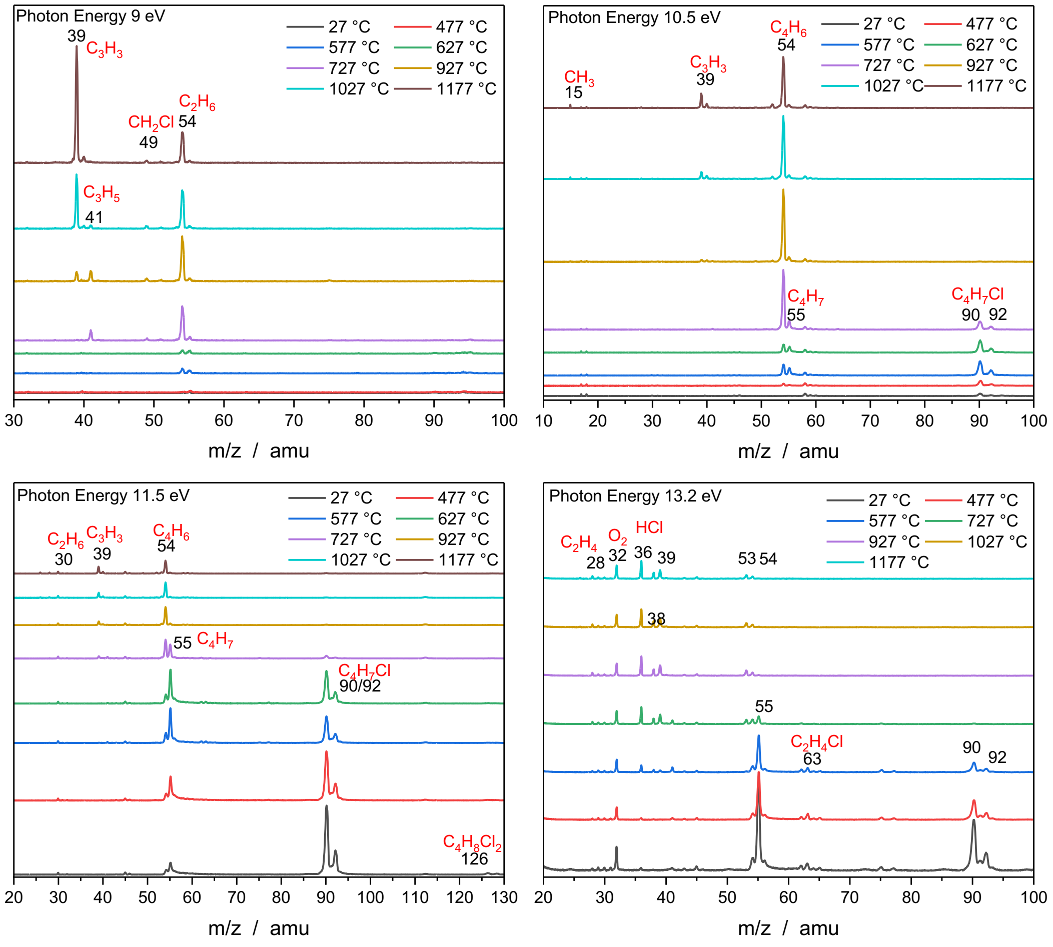

Figure S2 Mass spectra obtained from a Chen-type reactor during pyrolysis of 1,3-dichlorobutene (13DCB) at 9, 10.5, 11.5 and 13.2 eV, respectively, at different reactor temperatures. Conditions: 20 sccm 0.1% 13DCB in argon, 1mm iD Chen-type microreactor (*T* = RT–1200 °C, a), p_{inlet} = 10–20 mbar at a residence time of 20–50 μs. Intermediate and product detection using the operando PEPICO experiment at Swiss Light Source.

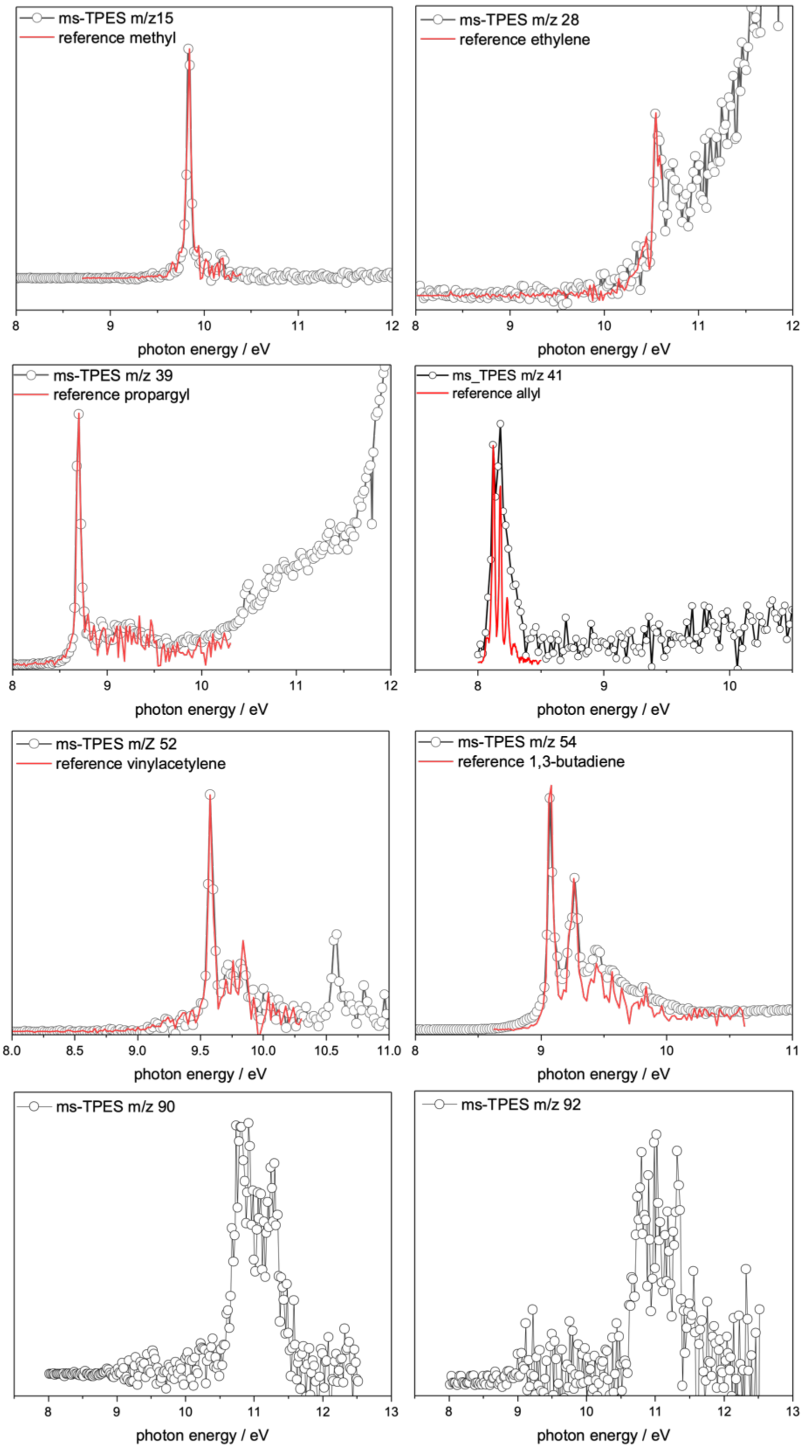

Figure S3 Photoion mass-selective threshold photoelectron spectra (ms-TPES) obtained during the pyrolysis of 13DCB in the Chen-type reactor. Reference spectra for methyl[17], ethylene, propargyl[17], allyl[18], vinylacetylene, and 1,3-butadiene[19] were obtained from the literature and downloaded from the PEPISCO database. Conditions: 20 sccm gas flow of 0.1% 13DCB in argon into the 1mm iD Chen-type microreactor (T = RT–1300 °C, a), p_{inlet} = 10–20 mbar at a residence time of 20–50 μs. Intermediate and product detection using the operando PEPICO experiment at Swiss Light Source.

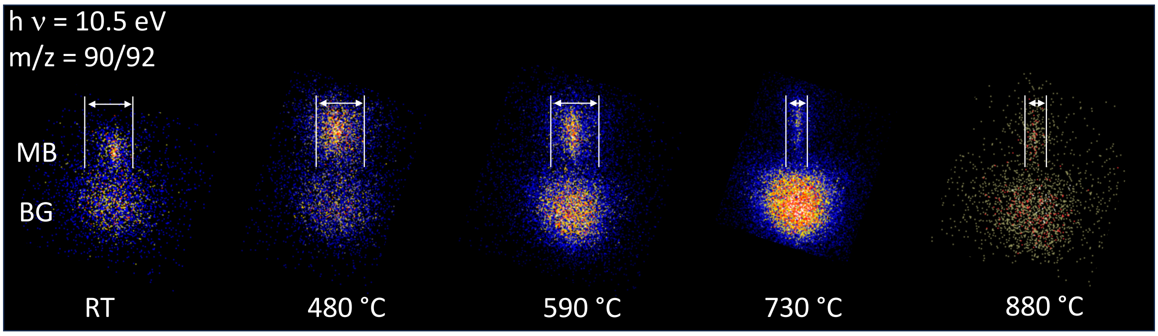

Figure S4 Ion velocity map images (VMI) of m/z 90/92 measured at 10.5 eV at different reactor temperatures. VMI enables us to distinguish between the background (BG) and the molecular beam (MB) component during pyrolysis, due to the fast expansion into vacuum. Fragmentation usually exhibits a broad horizontal component of the MB spot in the VMI, which is visible in the RT image (left), where 13DCB dissociatively ionizes to m/z 90/92 already at 10.5 eV. Upon heating 13DCB to 1000 K the MB spot gets narrower, indicative for a pyrolysis product at this mass under these conditions. We assign this species to C_4H_7Cl isomers, formed upon single dehydrochlorination of 13DCB. Conditions: 20 sccm gas flow of 0.1% 13DCB in argon into the 1mm iD Chen-type microreactor (T = RT–880 °C, p_{inlet} = 10–20 mbar at a residence time of 20–50 μs. Intermediate and product detection using the operando PEPICO experiment at Swiss Light Source.

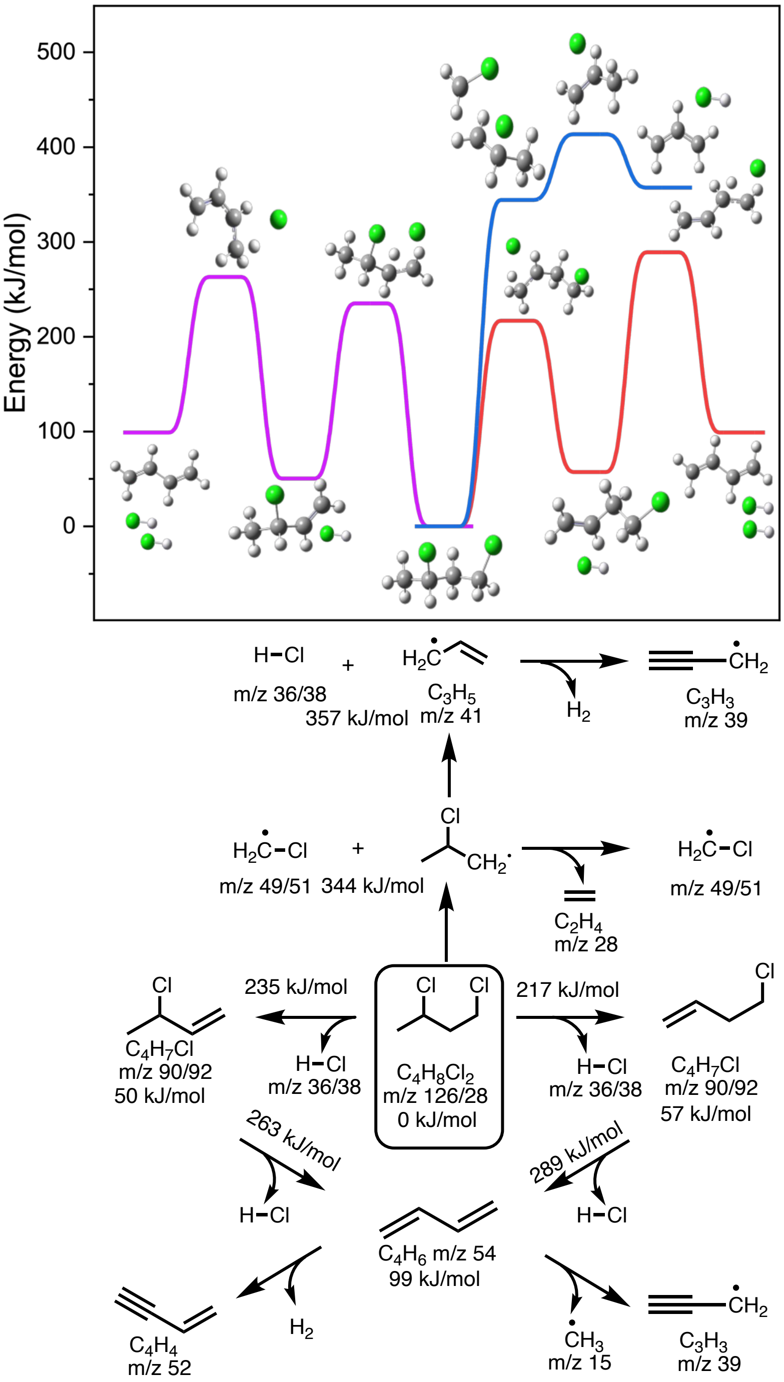

Figure S5 Upper trace: Potential energy surface of the 13DCB decomposition at G4 level of theory, based on the product observation in the microreactor. Two HCl elimination channels initiate the decomposition, leading to either 3-chloro1-butene and 4-chloro1-butene after passing a barrier of 235 and 217 kJ/mol, respectively. Both intermediate isomers (pink and red) can decompose in a second HCl loss channel (289 vs. 263 kJ/mol) to afford 1,3-butadiene at a product energy of 99 kJ/mol. Both reactions are proceeding in parallel, leading to 1,3-butadiene and 2 equivalents of HCl.

A third parallel reaction channel (blue), to afford chloromethyl radicals, opens after a transition state at 344 kJ/mol producing the 2-chloropropyl radical, which rapidly dehydrochlorinates to the allyl radical. Although the chloromethyl channel lies higher in energy, it is likely competitive with the initial dehydrochlorination. Chloropropyl radicals are subject to HCl loss forming allyl radical (357 kJ/mol) after a barrier of 414 kJ/mol. Lower part: Unimolecular chemistry of 1,3-butadiene: Butadiene cannot sustain higher reactor temperatures and decomposes further to methyl- and propargyl radicals, according to our observations. The radical formation proceeds after rearrangement to 1,2-butadiene, which was already investigated by Chambreau et al. using a similar reactor and photoionization mass spectrometry in conjunction with calculations.[20] In parallel, dehydrogenation of 1,3-butadiene is responsible for the formation of vinylacetylene (*m*/*z* 52). Allene and propyne (both *m*/*z* 40) as well as propargyl (*m*/*z* 39) are produced by allyl (*m*/*z* 41) dehydrogenation, while the minor products such as ethylene are formed either due to methyl recombination or rearrangement and CH_2Cl loss of the 2-chloropropyl radical.

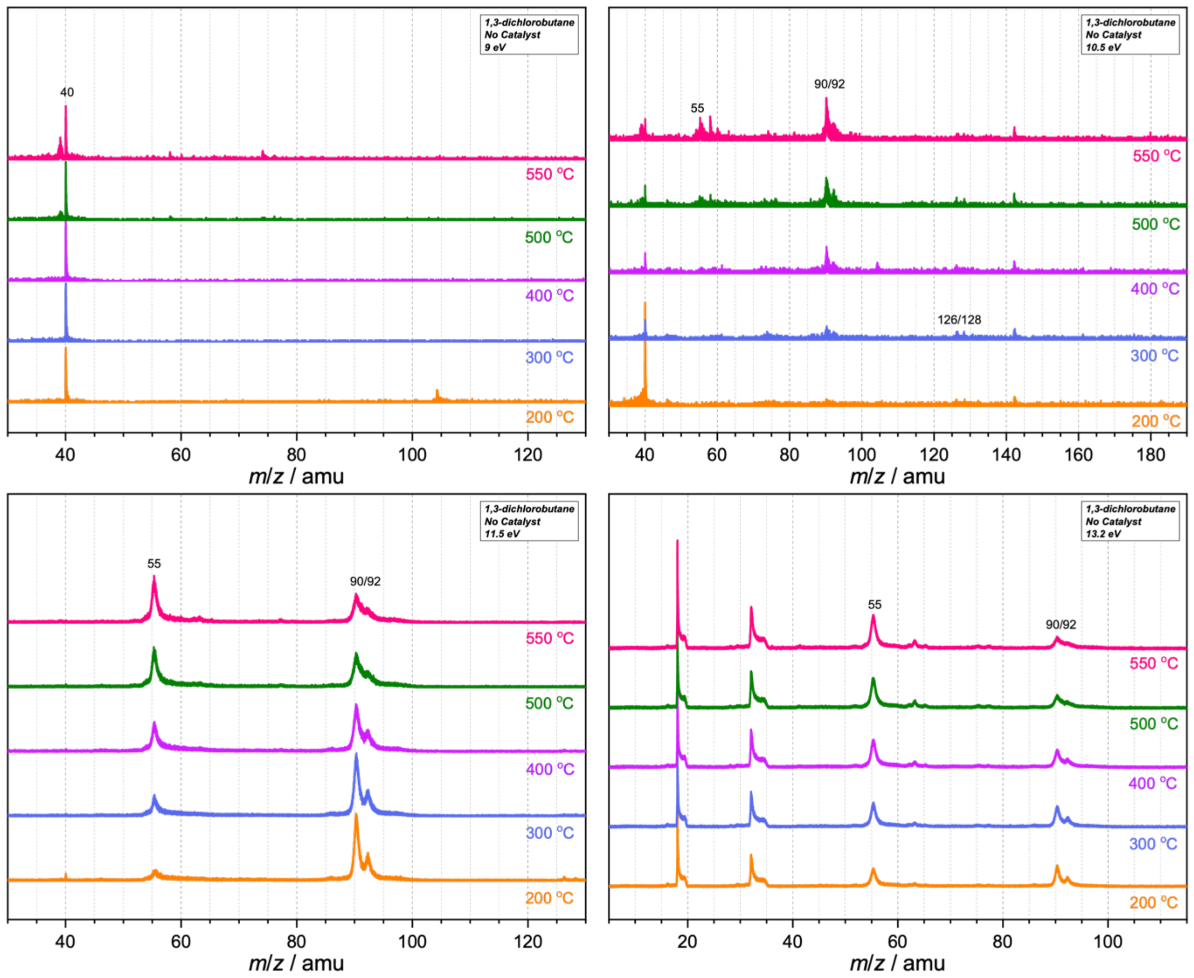

Figure S6 Mass spectra during the blank experiment, where 13DCB was lead over a heated bed of quartz glass wool. No product formation was observed. Conditions: 30 sccm gas flow of 0.3% 13DCB in argon into a quartz reactor equipped with 25 mg catalyst. Intermediate and product detection using the operando PEPICO experiment at Soleil Synchrotron. T = RT–550 °C, p_{inlet} = 0.3–1.3 bar.

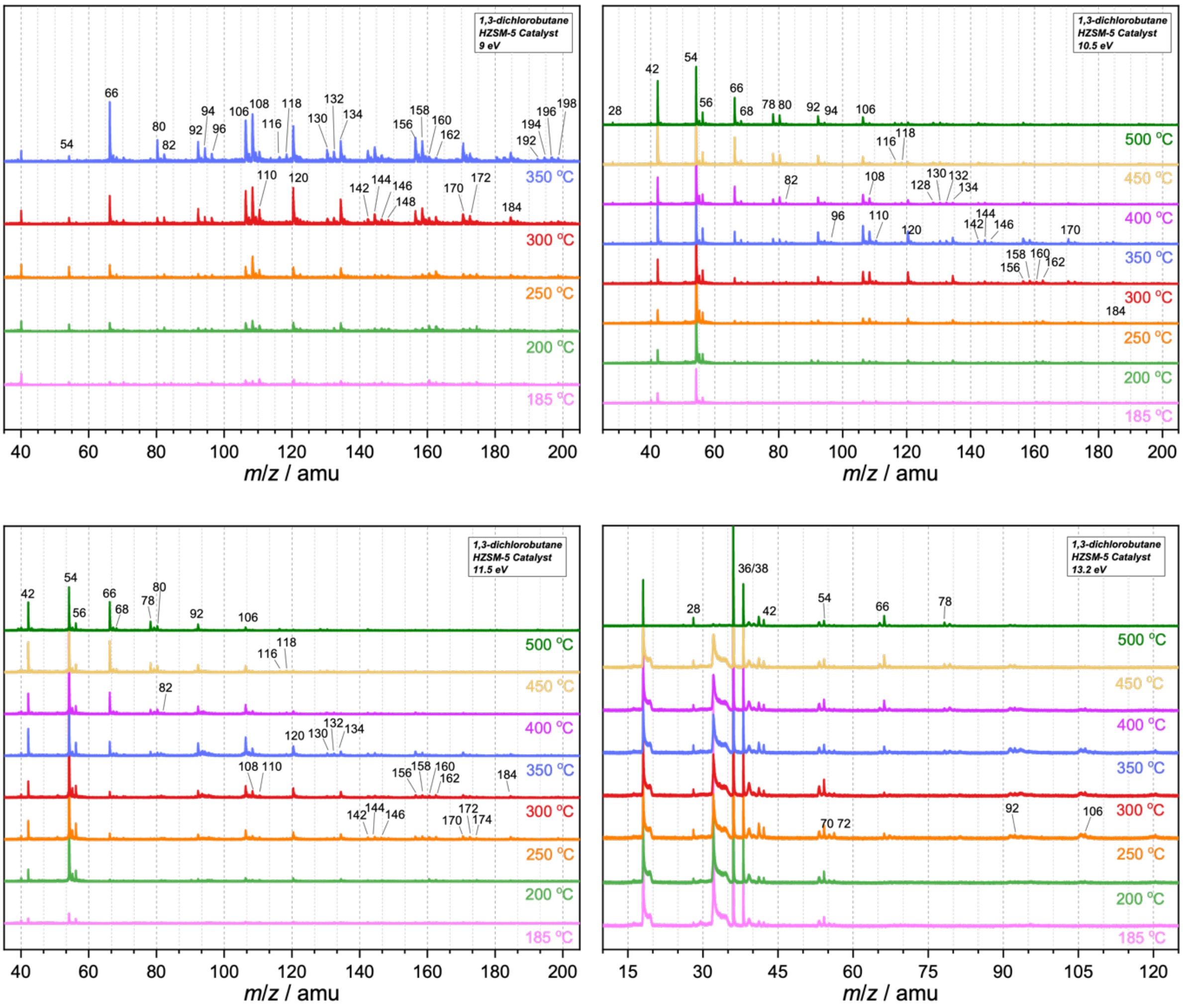

Figure S7 Mass spectra of 13DCB over a heated bed of HZSM-5. Conditions: 30 sccm gas flow of 0.3% 13DCB in argon in a quartz reactor filled with 25 mg catalyst. Intermediate and product detection using the operando PEPICO experiment at Soleil Synchrotron. T = RT–550 °C, p_{inlet} = 0.3–1.3 bar.

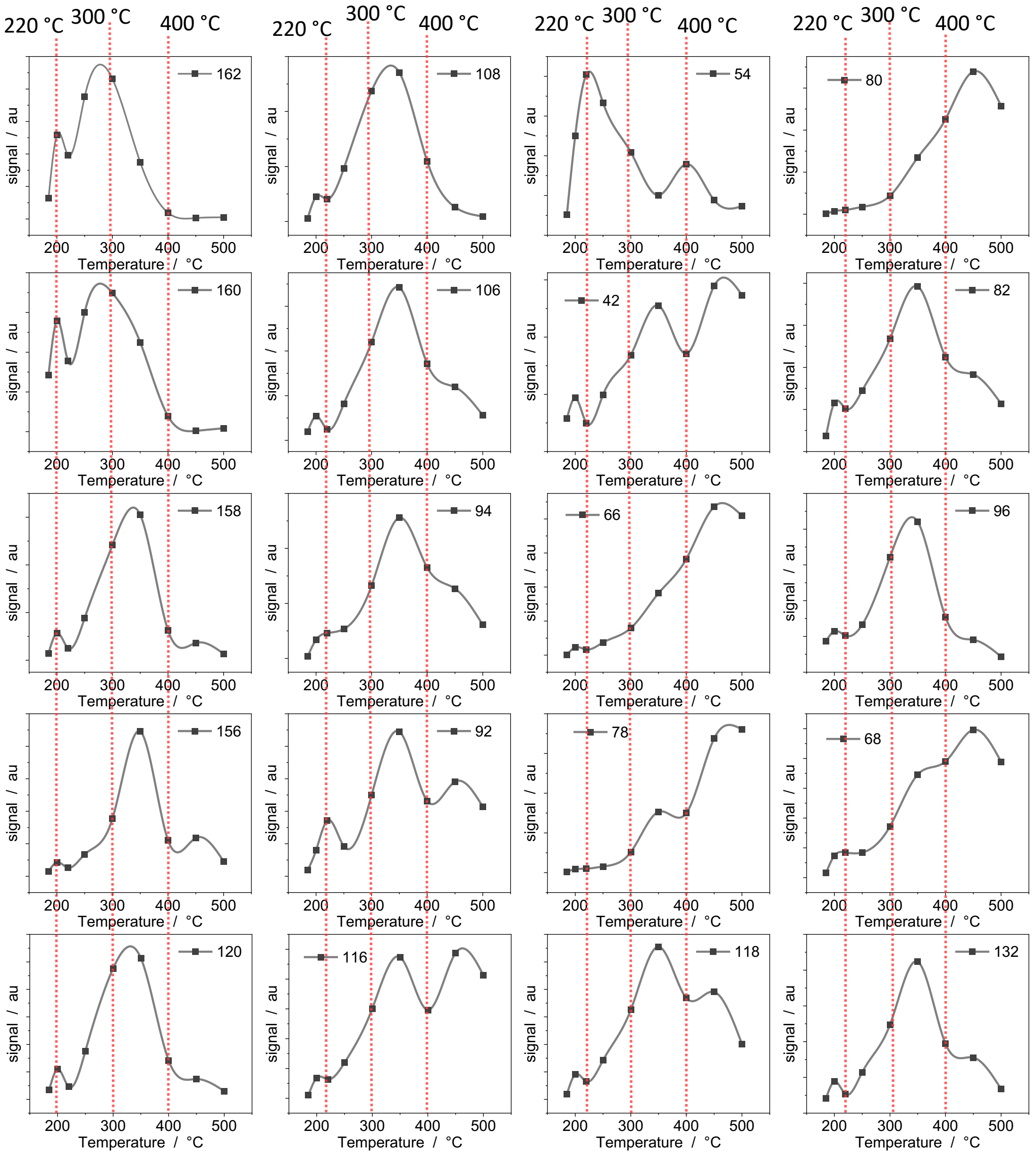

Figure S8 Integrated mass signals of 13DCB catalytic pyrolysis over HZSM-5 as function of the temperature. Conditions: 30 sccm gas flow of 0.3% 13DCB in argon into a quartz reactor equipped with 25 mg catalyst. Intermediate and product detection using the operando PEPICO experiment at Soleil Synchrotron. T = RT–550 °C, p_{inlet} = 0.3–1.2 bar.

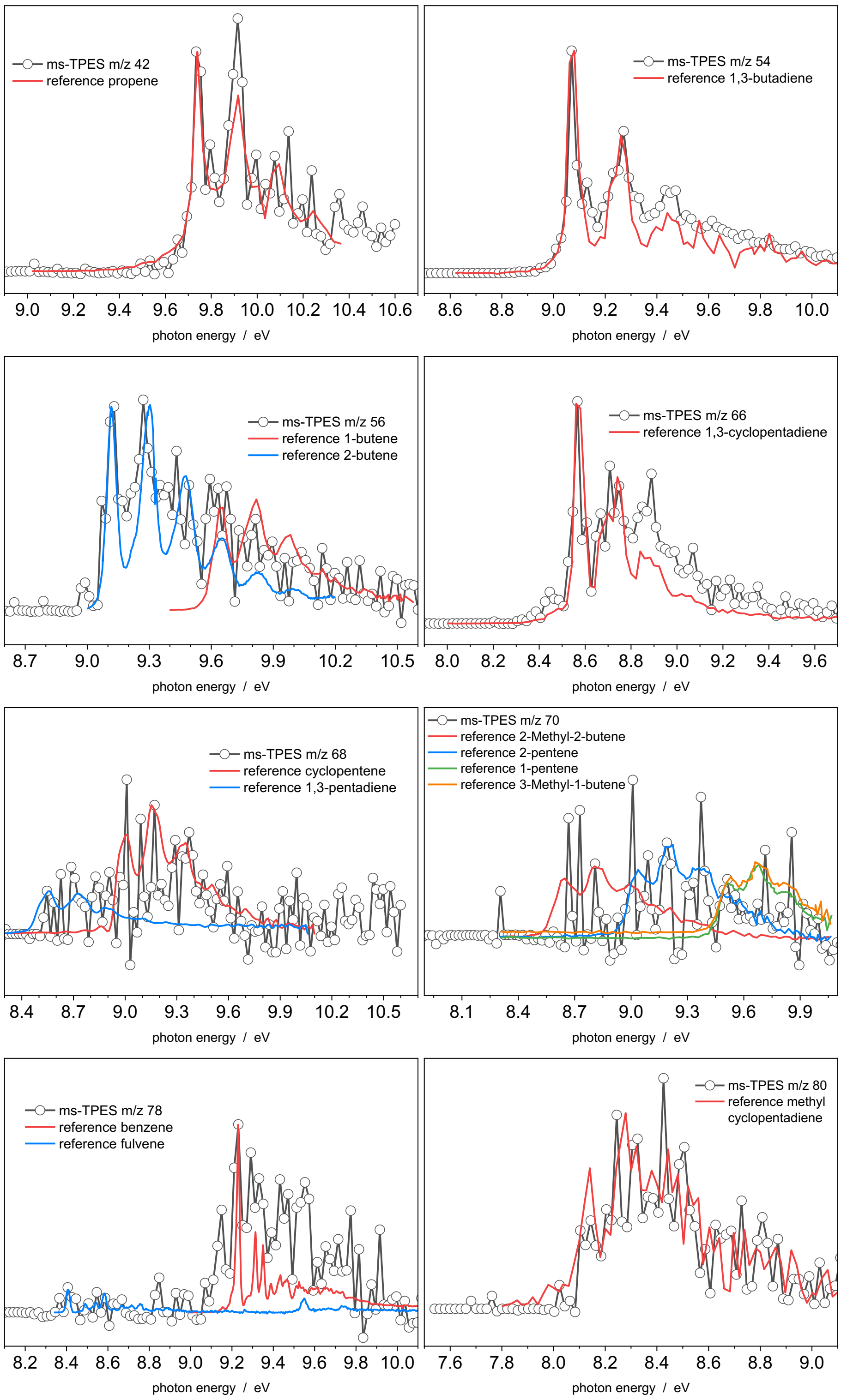

Figure S9 ms-TPES obtained during the catalytic pyrolysis of 13DCB over HZSM-5. In comparison with reference data: propene [21], 1,3-butadiene [19], 1- and 2-butene [22], 1,3-cyclopentadiene[19], cyclopentene[22], fulvene[23], benzene[23], and methylcyclopentadiene[17]. Franck–Condon simulations were carried out for cyclopentene, 1,3-pentadiene, methylbutenes, and pentenes. Conditions: 30 sccm gas flow of 0.3% 13DCB in argon into a quartz reactor equipped with 25 mg catalyst. Intermediate and product detection using the operando PEPICO experiment at Soleil Synchrotron. T = 300 °C, p_{inlet} = 0.3–1.2 bar.

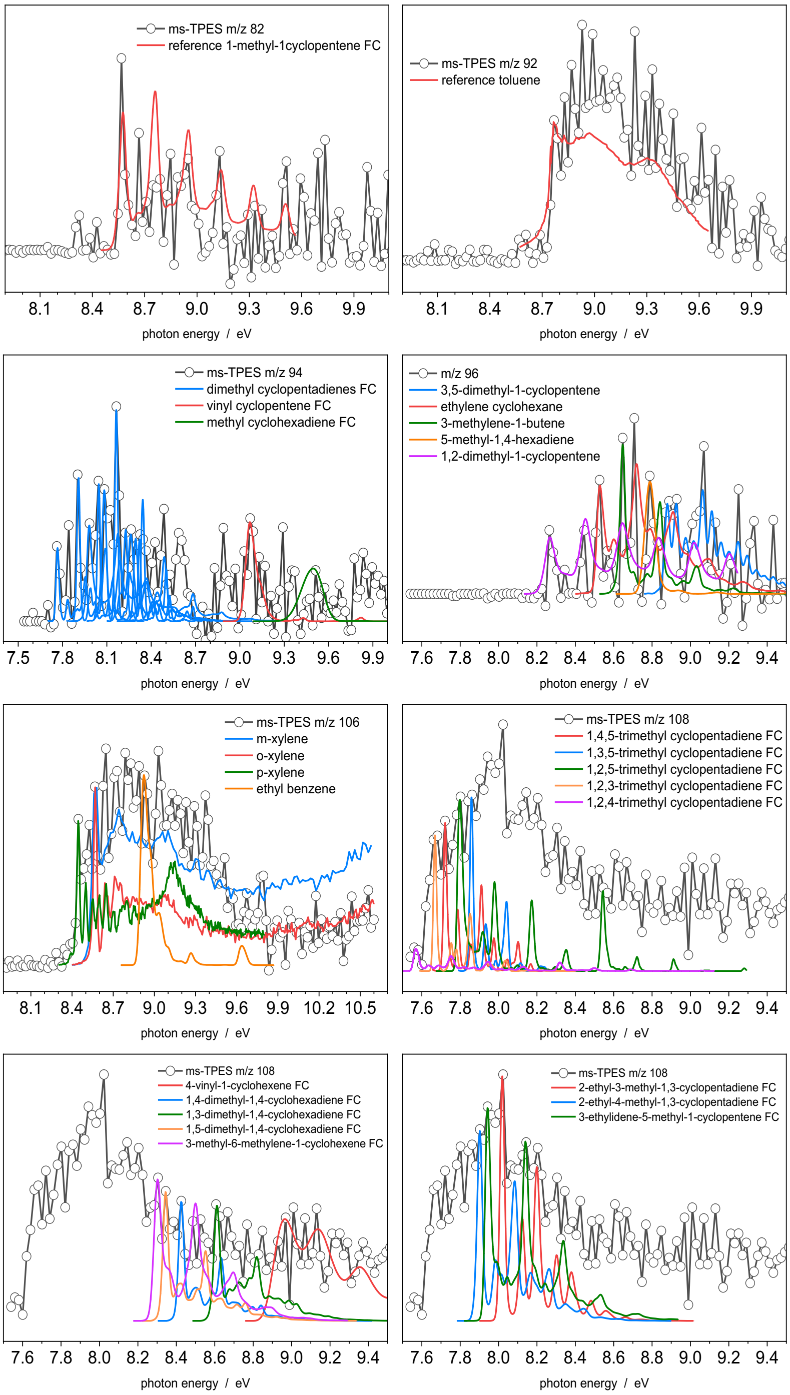

Figure S10 ms-TPES and PIE curves obtained during the catalytic pyrolysis of 13DCB over H-ZSM5. In comparison with reference data: toluene[24], and xylenes. Franck–Condon simulations include methylcyclopentane, dimethylcyclopentadienes, vinylcyclopentene, methylcyclohexadiene, dimethylcyclopentene, ethylenecyclohexane, methylenebutane, methylhexadiene, ethylbenzene, trimethylcyclopentadienes, vinylcyclohexadiene, dimethylcyclohexadienes, and ethylmethylcyclopentadienes. Conditions: 30 sccm gas flow of 0.3% 13DCB in argon into a quartz reactor equipped with 25 mg catalyst. Intermediate and product detection using the operando PEPICO experiment at Soleil Synchrotron. T = 300 °C, p_{inlet} = 0.3 – 1.2 bar.

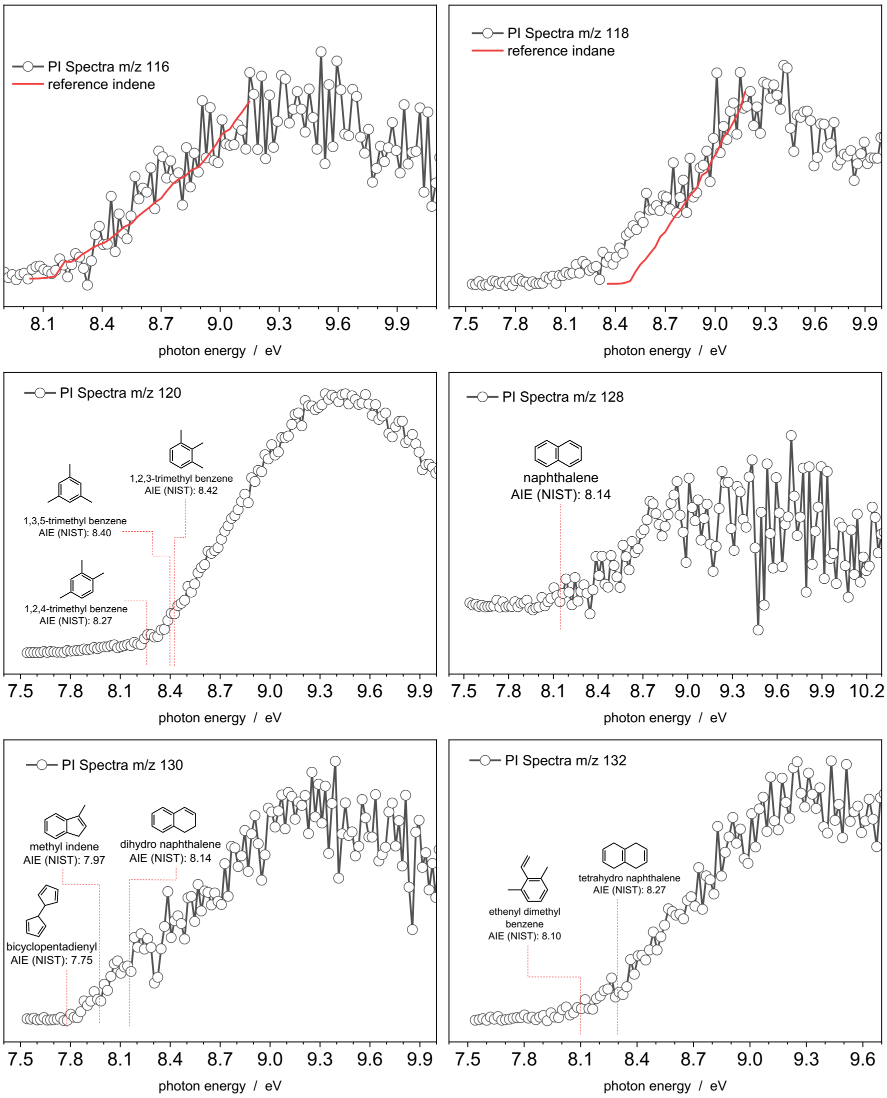

Figure S11 PI spectra obtained during the catalytic pyrolysis of 13DCB over HZSM5. In comparison with reference data: indene[25], and indane[25]. Reference adiabatic ionization energies including trimethyl benzenes[26], naphthalene[27], dihydronaphthalene[28], tetrahydronaphthalene[29]. Conditions: 30 sccm gas flow of 0.3% 13DCB in argon into a quartz reactor equipped with 25 mg catalyst. Intermediate and product detection using the operando PEPICO experiment at Soleil Synchrotron. T = 300 °C, p_{inlet} = 0.3 – 1.2 bar.

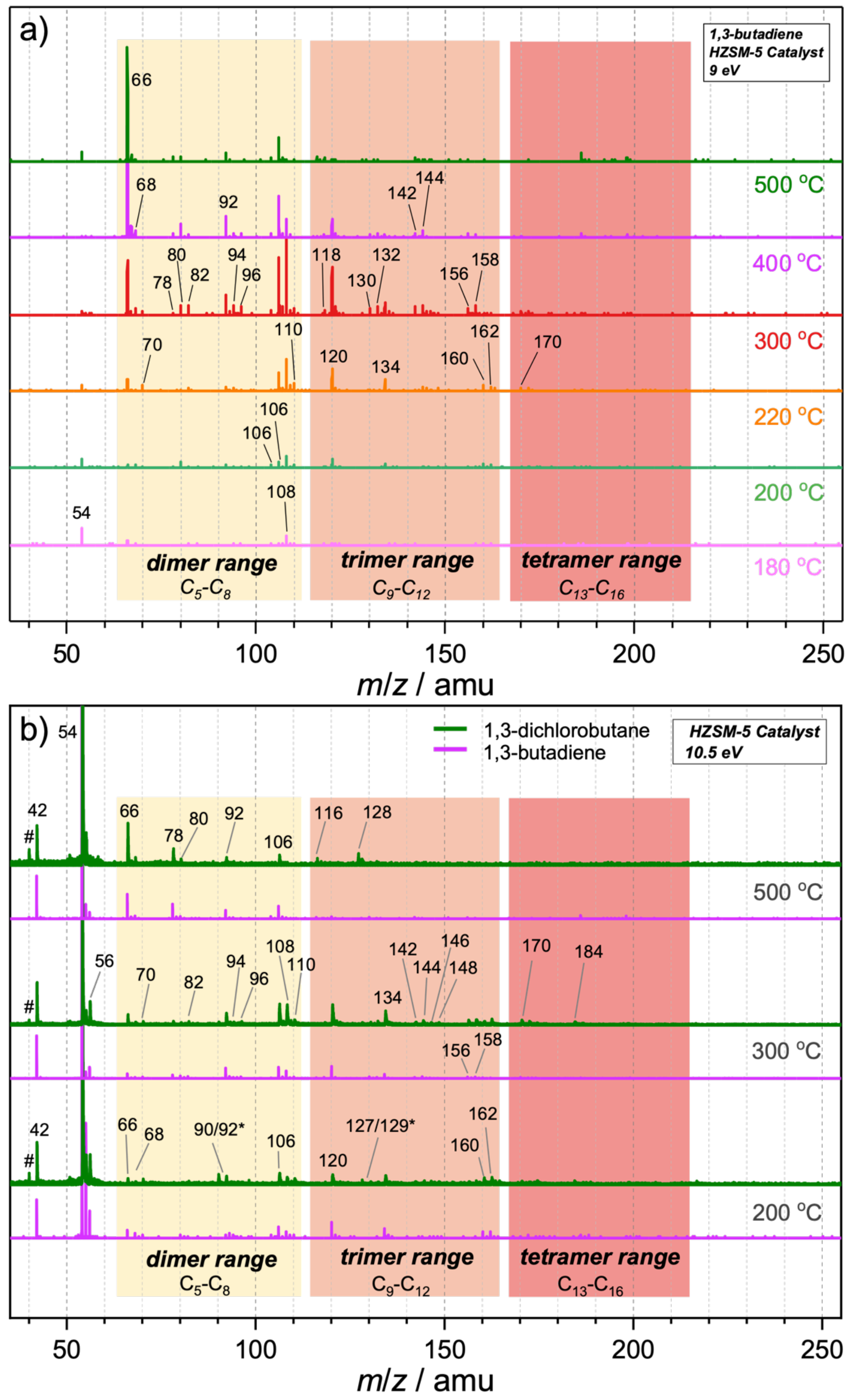

Figure S12 a) Mass spectra of the 1,3-butadiene reaction over HZSM-5, as measured at 9 eV at the Hefei Light Source. Conditions: 50 sccm gas flow of 0.1% 1,3-butadiene in argon into a quartz reactor equipped with 50 mg catalyst. Intermediate and product detection using the photoionization mass spectrometry setup at the National Synchrotron Radiation Lab (Hefei, China). T = 180–500 °C,

p_{inlet} = 7 mbar. **b**) Direct comparison of 1,3-butadiene and 13DCB (Figure S7, PEPICO detection). The light-off temperatures are similar at around 200 °C, showing initiation of oligomerization to afford *m*/*z* 106, 108, 120, 134, and 164. In particular, the large abundance of *m*/*z* 108 in the intermediate temperature range and the decomposition at higher temperatures are independent of the reactant used. These oligomeric species are converted to BTX at around 500 °C. Minor differences persist in the formation of *m*/*z* 90/92 (marked with an asterisk), which are attributed to chlorobutenes and are not present in case of butadiene. The peaks marked with # are attributed to ionization of argon due to higher order radiation.

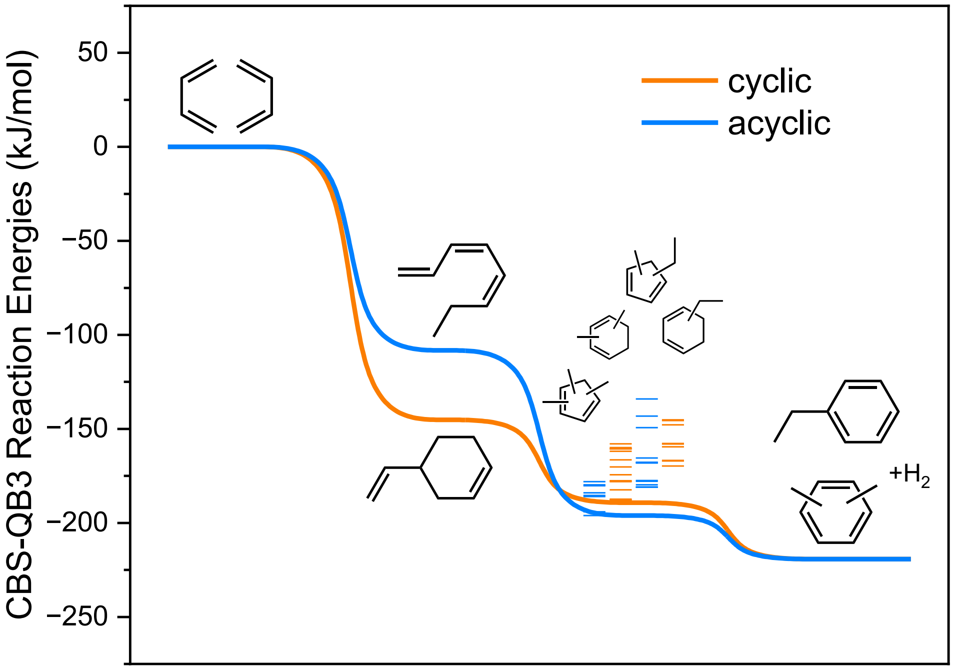

Figure S13 Butadiene dimerization pathway to *m*/*z* 108 isomers and hydrogen transfer reaction to xylene and ethylbenzene. The blue pathway (acyclic) resembles a head-to-head addition including an isomerization to 1,3,5-octatriene, while the orange pathway (cyclic) forms 3-vinylcyclohexene, the Diels–Alder product. Both pathways go downhill to 5- and 6-ring intermediates similar in energy, which reflect their easy isomerization via ring-expansion and contraction reactions. We have found more than 40 isomers within a 100 kJ/mol energy range, which can likely interconvert via ring-expansion and ring contraction reactions. A final hydrogen transfer reaction yields ortho-, meta- or para-xylene along with ethylbenzene, as observed (**Figure 2** in the main article).

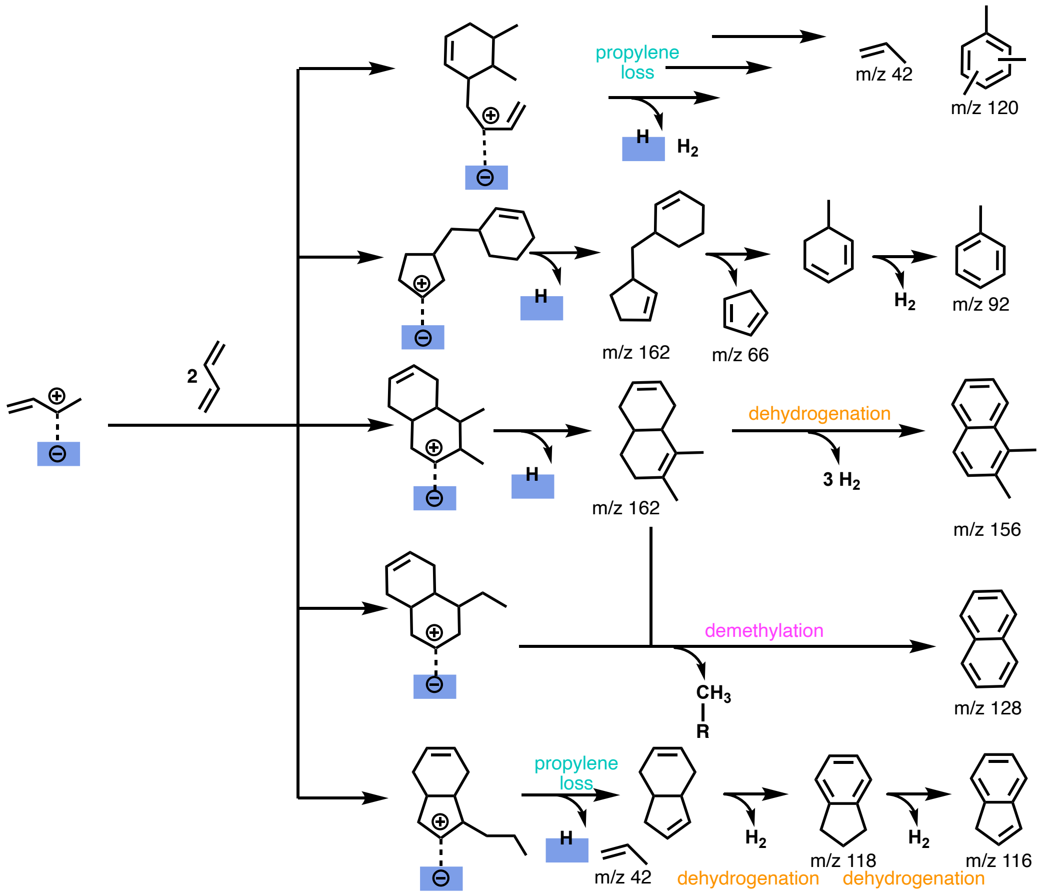

Figure S14 Anticipated butadiene trimer mechanisms over HZSM-5. Assumed surface species are color-coded in blue, while the black intermediates and products are observed after desorbtion from the catalyst. After butadiene trimerization, ring closure and rearrangement on the HZSM-5 surface, trimethylbenzenes (*m*/*z* 120) can be produced by a propylene loss, in agreement with the large abundance of both species in the mass spectrum at 350 °C (**Figure 2**). Analogously and due to the high concentration of cyclopentadiene, toluene may be formed in a similar reaction mechanism. Dimethylnaphthalenes (*m*/*z* 156, **Figure 2**) may be produced after a second ring closure reaction, from the observed *m*/*z* 162 intermediate, which exhibits maximum signal intensity at 300 °C (**Figure S8**) and dehydrogenates in a stepwise fashion affording *m*/*z* 156, while multiple methyl-transfer reactions produce naphthalene at *m*/*z* 128, formed with large abundance at 500 °C. Similarly, but via propylene loss, indene can be formed.

Table S1: Zero point (ZPE) corrected energies and adiabatic ionization energies of 47 *m*/*z* 108 isomers, calculated at CBS-QB3 level of theory. 2,3,4-trimethyl-1,3-cyclopentadiene is the lowest energy isomer.

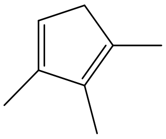	Isomer 1 – 2,3,4-trimethyl-1,3-cyclopentadiene<br>AIE in CBS-QB3 7.70 eV<br>Relative energy 0 kJ/mol
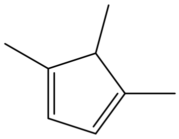	Isomer 2 - 1,4,5-trimethyl -1,3-cyclopentadiene<br>AIE in CBS-QB3 7.74 eV<br>Relative energy 11 kJ/mol
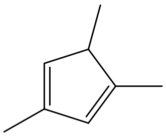	Isomer 3 - 2,4,5-trimethyl -1,3-cyclopentadiene<br>AIE in CBS-QB3 7.86 eV<br>Relative energy 10 kJ/mol
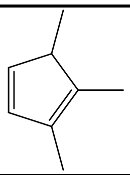	Isomer 4 - 1,2,5-trimethyl -1,3-cyclopentadiene<br>AIE in CBS-QB3 7.82 eV<br>Relative energy 12 kJ/mol
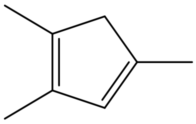	Isomer 5 - 1,2,4-trimethyl -1,3-cyclopentadiene<br>AIE in CBS-QB3 7.58 eV<br>Relative energy 2 kJ/mol
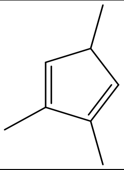	Isomer 6 - 2,3,5-trimethyl -1,3-cyclopentadiene<br>AIE in CBS-QB3 7.93 eV<br>Relative energy 16 kJ/mol
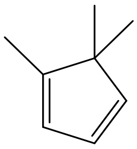	Isomer 7 - 1,5,5-trimethyl -1,3-cyclopentadiene<br>AIE in CBS-QB3 8.01 eV<br>Relative energy 16 kJ/mol
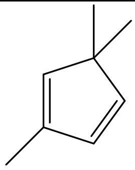	Isomer 8 - 2,5,5-trimethyl -1,3-cyclopentadiene<br>AIE in CBS-QB3 8.11 eV<br>Relative energy 18 kJ/mol
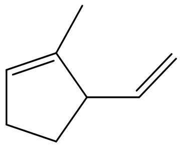	Isomer 9 - 1-methyl-5-vinyl-1-cyclopentene<br>AIE in CBS-QB3 8.65 eV<br>Relative energy 47 kJ/mol
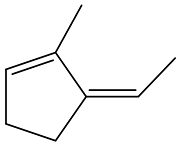	Isomer 10 – 5-ethylidene-1-methyl-1-cyclopentene<br>AIE in CBS-QB3 7.81 eV<br>Relative energy 15 kJ/mol

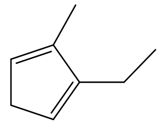	Isomer 11 – 2-ethyl-3-methyl-1,3-cyclopentadiene<br>AIE in CBS-QB3 8.02 eV<br>Relative energy 7 kJ/mol
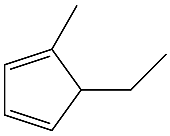	Isomer 12 – 5-ethyl-1-methyl-1,3-cyclopentadiene<br>AIE in CBS-QB3 8.04 eV<br>Relative energy 28 kJ/mol
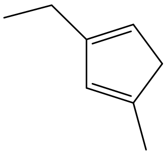	Isomer 13 – 2-ethyl-4-methyl-1,3-cyclopentadiene<br>AIE in CBS-QB3 7.90 eV<br>Relative energy 16 kJ/mol
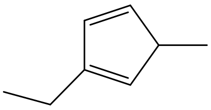	Isomer 14 – 3-ethyl-5-methyl-1,3-cyclopentadiene<br>AIE in CBS-QB3 8.16 eV<br>Relative energy 28 kJ/mol
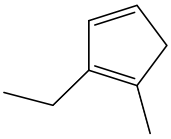	Isomer 15 – 2-ethyl-1-methyl-1,3-cyclopentadiene<br>AIE in CBS-QB3 7.86 eV<br>Relative energy 19 kJ/mol
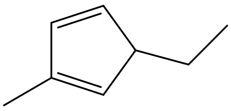	Isomer 16 – 5-ethyl-2-methyl-1,3-cyclopentadiene<br>AIE in CBS-QB3 8.16 eV<br>Relative energy 28 kJ/mol
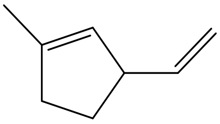	Isomer 17 – 1-methyl-3-vinyl-1-cyclopentene<br>AIE in CBS-QB3 8.63 eV<br>Relative energy 53 kJ/mol
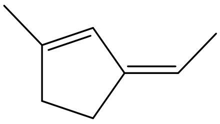	Isomer 18 – 3-ethylidene-1-methyl-1-cyclopentene<br>AIE in CBS-QB3 7.66 eV<br>Relative energy 15 kJ/mol
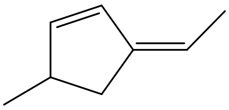	Isomer 19 - 3-ethylidene-5-methyl-1-cyclopentene<br>AIE in CBS-QB3 7.93 eV<br>Relative energy 31 kJ/mol
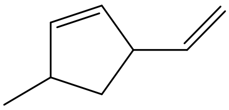	Isomer 20 - 3-methyl-5-vinyl-1-cyclopentene<br>AIE in CBS-QB3 8.99 eV<br>Relative energy 62 kJ/mol
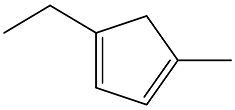	Isomer 21 - 1-ethyl-4-methyl-1,3-cyclopentadiene<br>AIE in CBS-QB3 7.76 eV<br>Relative energy 18 kJ/mol

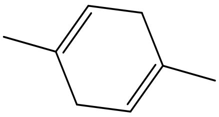	Isomer 22 – 1,4-dimethyl-1,4-cyclohexadiene<br>AIE in CBS-QB3 8.40 eV<br>Relative energy 9 kJ/mol
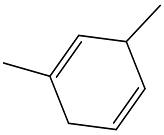	Isomer 23 – 1,3-dimethyl-1,4-cyclohexadiene<br>AIE in CBS-QB3 8.61 eV<br>Relative energy 19 kJ/mol
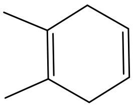	Isomer 24 – 1,2-dimethyl-1,4-cyclohexadiene<br>AIE in CBS-QB3 8.29 eV<br>Relative energy 10 kJ/mol
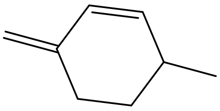	Isomer 25 – 3-methyl-6-methylene-1-cyclohexene<br>AIE in CBS-QB3 8.34 eV<br>Relative energy 18 kJ/mol
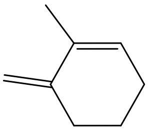	Isomer 26 – 2-methyl-3-methylene -1-cyclohexene<br>AIE in CBS-QB3 8.25 eV<br>Relative energy 14 kJ/mol
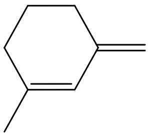	Isomer 27 – 1-methyl-3-methylene -1-cyclohexene<br>AIE in CBS-QB3 8.06 eV<br>Relative energy 0 kJ/mol
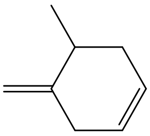	Isomer 28 – 4-methyl-5-methylene -1-cyclohexene<br>AIE in CBS-QB3 8.87 eV<br>Relative energy 36 kJ/mol
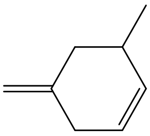	Isomer 29 – 3-methyl-5-methylene -1-cyclohexene<br>AIE in CBS-QB3 8.98 eV<br>Relative energy 34 kJ/mol
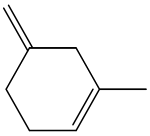	Isomer 30 – 2-methyl-4-methylene -1-cyclohexene<br>AIE in CBS-QB3 8.68 eV<br>Relative energy 26 kJ/mol
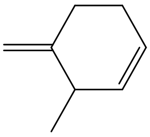	Isomer 31 – 3-methyl-4-methylene -1-cyclohexene<br>AIE in CBS-QB3 8.97 eV<br>Relative energy 38 kJ/mol
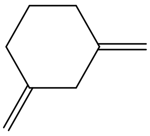	Isomer 32 – 1,3-dimethylene -1-cyclohexane<br>AIE in CBS-QB3 8.89eV<br>Relative energy 36 kJ/mol
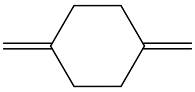	Isomer 33 – 1,4-dimethylene -1-cyclohexane<br>AIE in CBS-QB3 8.79 eV<br>Relative energy 35 kJ/mol

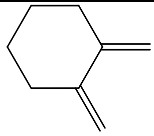	Isomer 34 – 1,2-dimethylene -1-cyclohexane<br>AIE in CBS-QB3 8.53 eV<br>Relative energy 35 kJ/mol
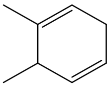	Isomer 35 – 3,4-dimethyl-1,4-cyclohexadiene<br>AIE in CBS-QB3 8.60 eV<br>Relative energy 22 kJ/mol
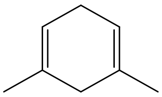	Isomer 36 – 2,4-dimethyl-1,4-cyclohexadiene<br>AIE in CBS-QB3 8.40 eV<br>Relative energy 8 kJ/mol
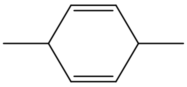	Isomer 37 – 3,6-dimethyl-1,4-cyclohexadiene<br>AIE in CBS-QB3 8.67 eV<br>Relative energy 30 kJ/mol
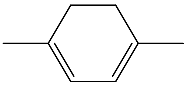	Isomer 38 – 1,4-dimethyl-1,3-cyclohexadiene<br>AIE in CBS-QB3 7.68 eV<br>Relative energy 7 kJ/mol
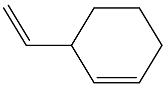	Isomer 39 – 3-vinyl-1-cyclohexene<br>AIE in CBS-QB3 8.98 eV<br>Relative energy 50 kJ/mol
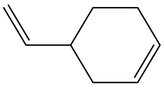	Isomer 40 – 4-vinyl-1-cyclohexene<br>AIE in CBS-QB3 9.03 eV<br>Relative energy 48 kJ/mol
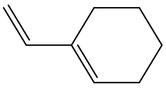	Isomer 41 – 1-vinyl-1-cyclohexene<br>AIE in CBS-QB3 8.26 eV<br>Relative energy 38 kJ/mol
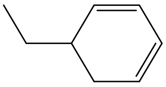	Isomer 42 – 6-ethyl-1,3-cyclohexadiene<br>AIE in CBS-QB3 8.18 eV<br>Relative energy 36 kJ/mol
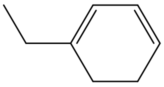	Isomer 43 – 1-ethyl-1,3-cyclohexadiene<br>AIE in CBS-QB3 7.91 eV<br>Relative energy 26 kJ/mol
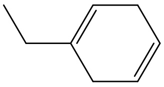	Isomer 44 – 1-ethyl-1,4-cyclohexadiene<br>AIE in CBS-QB3 8.58 eV<br>Relative energy 29 kJ/mol
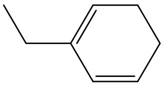	Isomer 45 – 2-ethyl-1,3-cyclohexadiene<br>AIE in CBS-QB3 8.00 eV<br>Relative energy 29 kJ/mol
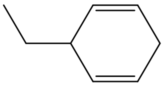	Isomer 46 – 3-ethyl-1,4-cyclohexadiene<br>AIE in CBS-QB3 8.60 eV<br>Relative energy 38 kJ/mol
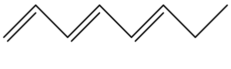	Isomer 47 – 1,3,5-octatriene<br>AIE in CBS-QB3 7.86 eV<br>Relative energy 88 kJ/mol

References:

[1] M. J. Frisch, G. W. Trucks, H. B. Schlegel, G. E. Scuseria, M. A. Robb, J. R. Cheeseman, G. Scalmani, V. Barone, G. A. Petersson, H. Nakatsuji, X. Li, M. Caricato, A. V. Marenich, J. Bloino, B. G. Janesko, R. Gomperts, B. Mennucci, H. P. Hratchian, J. V. Ortiz, A. F. Izmaylov, J. L. Sonnenberg, D. Williams-Young, F. Ding, F. Lipparini, F. Egidi, J. Goings, B. Peng, A. Petrone, T. Henderson, D. Ranasinghe, V. G. Zakrzewski, J. Gao, N. Rega, G. Zheng, W. Liang, M. Hada, M. Ehara, K. Toyota, R. Fukuda, J. Hasegawa, M. Ishida, T. Nakajima, Y. Honda, O. Kitao, H. Nakai, T. Vreven, K. Throssell, J. A. Montgomery, J. E. Peralta, F. Ogliaro, M. J. Bearpark, J. J. Heyd, E. N. Brothers, K. N. Kudin, V. N. Staroverov, T. A. Keith, R. Kobayashi, J. Normand, K. Raghavachari, A. P. Rendell, J. C. Burant, S. S. Iyengar, J. Tomasi, M. Cossi, J. M. Millam, M. Klene, C. Adamo, R. Cammi, J. W. Ochterski, R. L. Martin, K. Morokuma, O. Farkas, J. B. Foresman, D. J. Fox, **2019**, pp. Gaussian 16, Revision C.01.

[2] aL. A. Curtiss, P. C. Redfern, K. Raghavachari, *J. Chem. Phys.* **2007**, *126*, 084108; bJ. A. Montgomery, Jr., M. J. Frisch, J. W. Ochterski, G. A. Petersson, *J. Chem. Phys.* **2000**, *112*, 6532-6542.

[3] K. P. Somers, J. M. Simmie, *J. Phys. Chem. A* **2015**, *119*, 8922-8933.

[4] J. Bouwman, A. Bodi, P. Hemberger, *Phys. Chem. Chem. Phys.* **2018**, *20*, 29910-29917.

[5] C. Peng, H. Bernhard Schlegel, *Isr. J. Chem.* **2013**, *33*, 449-454.

[6] aM. Johnson, A. Bodi, L. Schulz, T. Gerber, *Nucl. Instrum. Methods Phys. Res. A* **2009**, *610*, 597-603; bB. Sztáray, K. Voronova, K. G. Torma, K. J. Covert, A. Bodi, P. Hemberger, T. Gerber, D. L. Osborn, *J. Chem. Phys.* **2017**, *147*, 013944-013944.

[7] B. Sztáray, T. Baer, *Rev. Sci. Instrum.* **2003**, *74*, 3763-3768.

[8] L. Nahon, N. de Oliveira, G. A. Garcia, J. F. Gil, B. Pilette, O. Marcouille, B. Lagarde, F. Polack, *J. Synchrotron Radiat.* **2012**, *19*, 508-520.

[9] Z. Zhang, J. Tian, X. Wu, I. Surin, J. Perez-Ramirez, P. Hemberger, A. Bodi, *J. Am. Chem. Soc.* **2023**, *145*, 7910-7917.

[10] aX. Tang, G. A. Garcia, J. F. Gil, L. Nahon, *Rev. Sci. Instrum.* **2015**, *86*, 123108; bG. A. Garcia, B. K. Cunha de Miranda, M. Tia, S. Daly, L. Nahon, *Rev. Sci. Instrum.* **2013**, *84*, 053112.

[11] Z. Zhou, X. Du, J. Yang, Y. Wang, C. Li, S. Wei, L. Du, Y. Li, F. Qi, Q. Wang, *J. Synchrotron. Radiat.* **2016**, *23*, 1035-1045.

[12] Z. Zhou, J. Yang, W. Yuan, Z. Wang, Y. Pan, F. Qi, *Phys. Chem. Chem. Phys.* **2022**, *24*, 21567-21577.

[13] X. Guo, G. Fang, G. Li, H. Ma, H. Fan, L. Yu, C. Ma, X. Wu, D. Deng, M. Wei, D. Tan, R. Si, S. Zhang, J. Li, L. Sun, Z. Tang, X. Pan, X. Bao, *Science* **2014**, *344*, 616-619.

[14] D. Felsmann, K. Moshammer, J. Krüger, A. Lackner, A. Brockhinke, T. Kasper, T. Bierkandt, E. Akyildiz, N. Hansen, A. Lucassen, P. Oßwald, M. Köhler, G. A. Garcia, L. Nahon, P. Hemberger, A. Bodi, T. Gerber, K. Kohse-Höinghaus, *Proc. Combust. Inst.* **2015**, *35*, 779-786.

[15] aS. Grimm, S.-J. Baik, P. Hemberger, A. Bodi, A. M. Kempf, T. Kasper, B. Atakan, *Phys. Chem. Chem. Phys.* **2021**, *23*, 15059-15075; bQ. Guan, K. N. Urness, T. K. Ormond, D. E. David, G. Barney Ellison, J. W. Daily, *Int. Rev. Phys. Chem.* **2014**, *33*, 447-487.

[16] Z. Pan, A. Puente-Urbina, S. R. Batool, A. Bodi, X. Wu, Z. Zhang, J. A. van Bokhoven, P. Hemberger, *Nat. Commun.* **2023**, *14*, 4512.

[17] N. Lokachari, G. Kukkadapu, B. D. Etz, G. M. Fioroni, S. Kim, M. Steglich, A. Bodi, P. Hemberger, S. S. Matveev, A. Thomas, H. Song, G. Vanhove, K. Zhang, G. Dayma, M. Lailliau, Z. Serinyel, A. A. Konnov, P. Dagaut, W. J. Pitz, H. J. Curran, *Combust. and Flame* **2023**, *251*, 112547.

[18] P. Hemberger, X. Wu, Z. Pan, A. Bodi, *J. Phys. Chem. A* **2022**, *126*, 2196-2210.

[19] Z. Pan, A. Puente-Urbina, A. Bodi, J. A. van Bokhoven, P. Hemberger, *Chem. Sci.* **2021**, *12*, 3161-3169.

[20] S. D. Chambreau, J. Lemieux, L. Wang, J. Zhang, *J. Phys. Chem. A* **2005**, *109*, 2190-2196.

[21] X. Wu, Z. Zhang, Z. Pan, X. Zhou, A. Bodi, P. Hemberger, *Angew. Chem. Int. Ed.* **2022**, *61*, e202207777.

[22] J. Pieper, S. Schmitt, C. Hemken, E. Davies, J. Wullenkord, A. Brockhinke, J. Krüger, G. A. Garcia, L. Nahon, A. Lucassen, W. Eisfeld, K. Kohse-Höinghaus, *Zeitschr. Phys. Chem.* **2018**, *232*, 153-187.
[23] J. D. Savee, B. Sztáray, P. Hemberger, J. Zádor, A. Bodi, D. L. Osborn, *Faraday Discussions* **2022**, *238*, 645-664.
[24] T. P. Debies, J. W. Rabalais, *J. Electron Spectros. Relat. Phenomena* **1972**, *1*, 355-370.
[25] Z. Zhou, M. Xie, Z. Wang, F. Qi, *Rapid Commun. Mass Spectrom.* **2009**, *23*, 3994-4002.
[26] aJ. O. Howell, J. M. Goncalves, C. Amatore, L. Klasinc, R. M. Wightman, J. K. Kochi, *J. Am. Chem. Soc.* **1984**, *106*, 3968-3976; bS. G. Lias, P. Ausloos, *J. Am. Chem. Soc.* **1978**, *100*, 6027-6034.
[27] M. C. R. Cockett, H. Ozeki, K. Okuyama, K. Kimura, *J. Chem. Phys.* **1993**, *98*, 7763-7772.
[28] C. Dass, M. L. Gross, *J. Am. Chem. Soc.* **1983**, *105*, 5724-5729.
[29] E. Heilbronner, F. Brogli, E. Vogel, *J. Electron Spectros. Relat. Phenomena* **1976**, *9*, 227-239.
